# Chalcogen-Bonded [Se–N]_2_ Cyclic Supramolecular Synthons Enhanced by Halogen Bonds: Studies in the Gas Phase and Crystalline Phase

**DOI:** 10.3390/ijms26052324

**Published:** 2025-03-05

**Authors:** Shaobin Miao, Xiaotian Sun, Yu Zhang, Weizhou Wang

**Affiliations:** College of Chemistry and Chemical Engineering, and Henan Key Laboratory of Function-Oriented Porous Materials, Luoyang Normal University, Luoyang 471934, China; miaoshaobin@126.com (S.M.); sxt@lynu.edu.cn (X.S.); yzhpaper@yahoo.com (Y.Z.)

**Keywords:** supramolecular synthon, chalcogen bond, halogen bond, PBE0-D3 calculations, cocrystal structures

## Abstract

Chalcogen-bonded [Se–N]_2_ is a strong cyclic supramolecular synthon in supramolecular chemistry. Selenadiazole is commonly used in the synthesis of [Se–N]_2_. One nitrogen atom in a selenadiazole molecule participates in the formation of [Se–N]_2_, while the other nitrogen atom can participate in the formation of other types of noncovalent bonds. Investigating the effect of neighboring noncovalent bonds on [Se–N]_2_ is beneficial for its further synthesis and application. In this study, we combined theoretical calculations and crystallography to explore the effect of I···N halogen bonds on [Se–N]_2_ in both the gas phase and the crystalline phase. Gas-phase calculations show that the formation of halogen bonds increases the strength of [Se–N]_2_, and the strength of the halogen bond is directly proportional to the strength of [Se–N]_2_. In the crystalline phase, [Se–N]_2_ is influenced by more noncovalent bonds in addition to halogen bonds, making the results more complex. However, if the effect of other noncovalent bonds is relatively small, the strength of the halogen bond remains directly proportional to the strength of [Se–N]_2_. It is believed that the conclusions drawn from halogen bonds are also applicable to other types of noncovalent bonds.

## 1. Introduction

It is well known that hydrogen bonds are the simplest yet most important type of noncovalent bonds in nature. In recent years, in addition to hydrogen bonds, numerous studies have shown that other types of noncovalent bonds, such as halogen bonds and chalcogen bonds, are becoming more relevant in areas like crystal engineering, molecular recognition, drug design, catalysis and self-assembly in materials science, where directional and specific interactions are needed to create stable molecular complexes [1,2,3,4]. Similar to hydrogen bonds, the International Union of Pure and Applied Chemistry (IUPAC) has also recommended definitions for halogen and chalcogen bonds [2,4]. In 2007, Clark et al. introduced the σ-hole concept based on halogen bonds [5]. Later, Murray and colleagues expanded on this concept, successfully explaining the formation of other types of noncovalent bonds [6]. In recent years, the σ-hole concept has been widely accepted and applied in various research fields related to noncovalent bonds [7,8,9,10,11]. Both halogen bonds and chalcogen bonds are subsets of σ-hole bonds.

**Figure 1:**
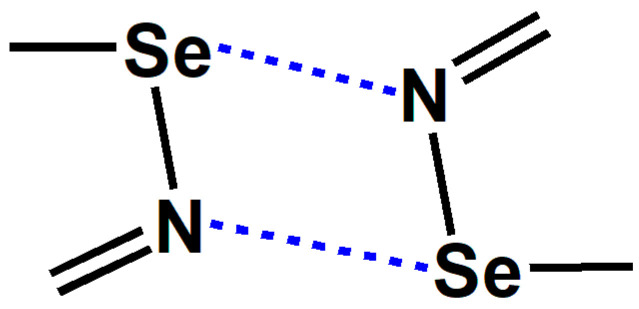
The chalcogen-bonded [Se–N]_2_ cyclic supramolecular synthon.

The [Se–N]_2_ in Figure 1 is a cyclic supramolecular synthon assembled from two Se···N chalcogen bonds. The assembly of the two Se···N chalcogen bonds in [Se–N]_2_ is stronger than a single Se···N chalcogen bond and has greater potential for applications. The chalcogen-bonded [Se–N]_2_ cyclic supramolecular synthon has been used to synthesize many supramolecular architectures with special structures and properties [12,13,14,15,16,17,18,19,20]. One impressive work is the synthesis of a series of supramolecular capsules by Yu and coworkers using the [Se–N]_2_ supramolecular synthons [20]. The supramolecular capsules are very useful because Rebek’s extensive previous work has shown that the chemistry inside supramolecular capsules is very fascinating and exciting [21]. Other important work focuses on utilizing the [Se–N]_2_ supramolecular synthons to construct organic small-molecule or polymer optoelectronic materials with good performance [12,15,16]. A search in the Cambridge Structure Database (CSD version 5.45) shows that there are 184 crystal structures that include the [Se–N]_2_ supramolecular synthons [22,23]. Given the growing interest in chalcogen bonds, we believe that such crystal structures will become more prevalent in the future. At least in this work, we have synthesized five new cocrystals containing the [Se–N]_2_ supramolecular synthon.

In addition to the experimental investigation, there are also computational studies on [Se–N]_2_ supramolecular synthons [24,25,26,27,28]. Lu’s group and Wang’s group conducted detailed computational studies on the effect of different substituents on [Se–N]_2_ supramolecular synthons [24,27]. If selenadiazole molecules are used to construct [Se–N]_2_ supramolecular synthons, each selenadiazole molecule contains an additional nitrogen atom that can form noncovalent bonds, and this scenario is commonly observed in the crystal structures. Therefore, studying the effect of noncovalent bonds on [Se–N]_2_ supramolecular synthons is just as important as studying the effect of substituents on [Se–N]_2_ supramolecular synthons. On the other hand, studying the cooperativity and competition between different types of noncovalent bonds has long been one of the core aspects of crystal engineering. The cooperativity and competition between halogen bonds and the following noncovalent interactions have been well-documented: anion-π interactions, cation-π interactions, π-π stacking interactions, lone pair-π interactions, other halogen bonds, and tetrel bonds [29,30,31,32,33,34]. In this study, we investigated the effect of I···N halogen bonds on the [Se–N]_2_ supramolecular synthons in both the gas phase and the crystalline state. We selected 2,1,3-benzoselenadiazole (BSeD) as a representative molecule of selenium-containing diazoles. This is mainly due to two reasons: (1) the complex formed by BSeD molecules and other small molecules is relatively small, making it suitable for high-precision quantum chemical calculations, and (2) BSeD can easily form cocrystals with other iodine-containing molecules, making it an ideal molecule for crystallographic studies. Furthermore, in order to test the generality of the conclusions drawn from the [Se–N]_2_ supramolecular synthons formed by BSeD, we also studied the [Se–N]_2_ supramolecular synthons formed by the fluorine-substituted BSeD, namely 5-fluoro-2,1,3-benzoselenadiazole (F-BSeD).

**Figure 2:**
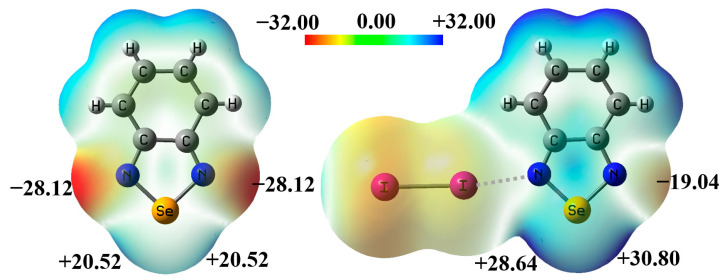
The electrostatic potential (in kcal/mol) maps of BSeD and I_2_···BSeD. The values represent the locally most positive or most negative electrostatic potential.

Figure 2 shows the electrostatic potential maps of the monomer BSeD and the dimer I_2_···BSeD calculated at the PBE0-D3/def2-TZVPP level of theory [35,36,37]. One Se atom in a BSeD molecule has two σ-holes [38]. In Figure 2, the negative electrostatic potential value represents the local minimum in the lone pair electron region of the N atom, and the positive electrostatic potential value represents the local maximum in the σ-hole region of the Se atom. The geometries of BSeD and I_2_···BSeD were not optimized and taken directly from the crystal structure of the cocrystal between I_2_ and BSeD (CSD refcode GAJQUG) [14]. By comparing the electrostatic potential maps of BSeD and I_2_···BSeD in Figure 2, it can be seen that the formation of the I···N halogen bond causes the electrostatic potential at the regions of the two σ-holes of the Se atom and the lone pair electrons of the free N atom to become more positive. This aligns with general chemical intuition, as the formation of the I···N halogen bond leads to electron density shifting from the lone pair electrons of the N atom towards the σ* anti-bonding orbitals of the I–I bond. In other words, the formation of the I···N halogen bond is equivalent to the effect of an electron-withdrawing substituent. However, the increase in the electrostatic potential at the region of the Se atom’s σ-hole enhances the Se···N chalcogen bond, while the increase in the electrostatic potential at the region of the lone pair electrons of the N atom weakens the Se···N chalcogen bond. Therefore, under the effect of the I···N halogen bond, whether the [Se–N]_2_ cyclic supramolecular synthon becomes stronger or weaker remains an open question that requires further investigation. This is also the most important issue that this study aims to address in both the gas phase and the crystalline state.

## 2. Results and Discussion

### 2.1. The Effect of Halogen Bonds on the [Se–N]_2_ Supramolecular Synthons in the Gas Phase

**Figure 3:**
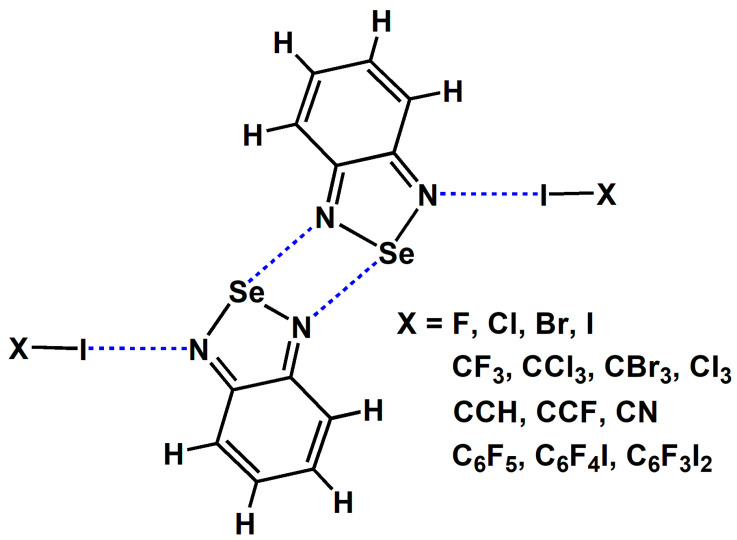
The chemical structures of the tetramers studied in this study.

We did not conduct experiments related to the gas phase; instead, we used the data from gas-phase quantum chemical calculations to replace the experimental data from the gas phase. Figure 3 shows the chemical structures of the tetramers studied in this study. The two I···N halogen bonds and two Se···N chalcogen bonds are both symmetrical. In Figure 3, the molecular formula C_6_F_4_I_2_ represents the 1,4-diiodotetrafluorobenzene molecule, and C_6_F_3_I_3_ represents the 1,3,5-trifluoro-2,4,6-triiodobenzene molecule. Except for C_6_F_4_I_2_ and C_6_F_3_I_3_, all the other substances are common small molecules, and their names can be inferred directly from their molecular formulas. The I···N halogen bonds in Figure 3 can be divided into four types: one type is the I···N halogen bonds formed by diatomic halogen molecules; another type is the C(*sp*^3^)–I···N halogen bond; the third type is the C(*sp*^2^)–I···N halogen bond; and the fourth type is the C(*sp*)–I···N halogen bond. These four types of halogen bonds are widely representative.

**Figure 4:**
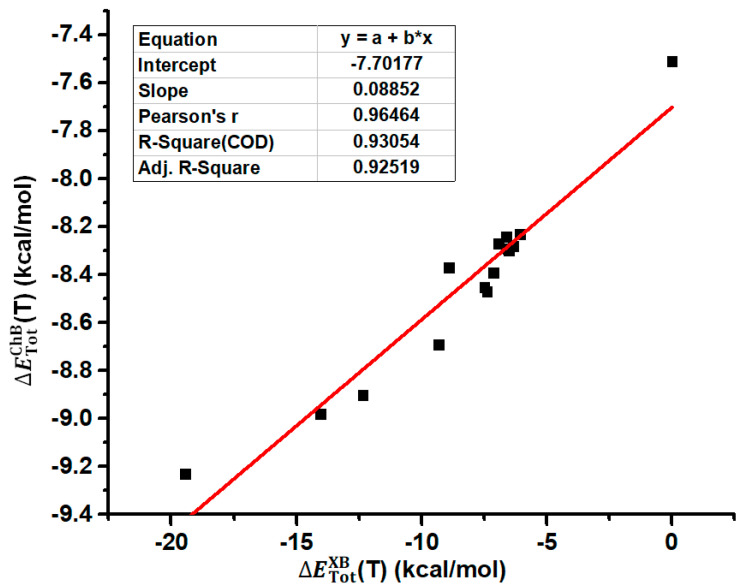
Correlation between ${∆E}_{Tot}^{XB}$(T) and ${∆E}_{Tot}^{ChB}$(T).

**Figure 5:**
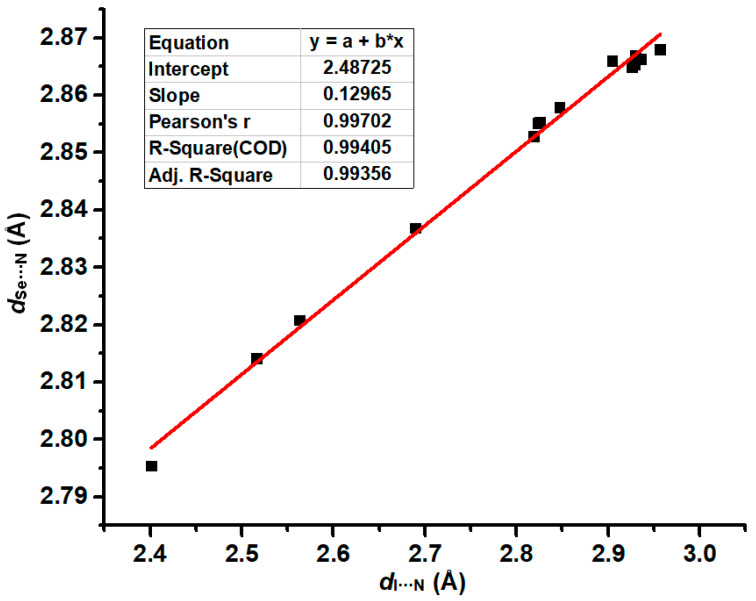
Correlation between *d*_I···N_ and *d*_Se···N_.

The structures of all the tetramers in Figure 3 were fully optimized at the PBE0-D3/def2-TZVPP level of theory. Table 1 summarizes the interatomic distances *d*_I···N_ and *d*_Se···N_ in the tetramers, the total interaction energies of the halogen bonds in the tetramers, the total interaction energies of the chalcogen bonds in the tetramers and BSeD···BSeD dimers, and the differences between ${∆E}_{Tot}^{ChB}$(T) and ${∆E}_{Tot}^{ChB}$(D) calculated at the PBE0-D3/def2-TZVPP level of theory. Here, the chalcogen bond refers to the [Se–N]_2_ cyclic supramolecular synthon, and actually, it contains two single Se···N chalcogen bonds. Evidently, the chalcogen bonds in the tetramers are influenced by the I···N halogen bonds. Therefore, the difference between ${∆E}_{Tot}^{ChB}$(T) and ${∆E}_{Tot}^{ChB}$(D) clearly reflects the effect of the I···N halogen bonds on the [Se–N]_2_ supramolecular synthons. According to the values of ${∆E}_{Tot}^{ChB}$(T)−${∆E}_{Tot}^{ChB}$(D) in Table 1, the formation of I···N halogen bonds significantly strengthens the [Se–N]_2_ supramolecular synthons. Figure 4 shows the correlation between ${∆E}_{Tot}^{XB}$(T) and ${∆E}_{Tot}^{ChB}$(T). The total interaction energies of the I···N halogen bonds in the tetramers are positively correlated with the total interaction energies of the [Se–N]_2_ cyclic supramolecular synthons in the tetramers, indicating that as the halogen bonds gradually strengthen, the [Se–N]_2_ cyclic supramolecular synthons also gradually strengthen. The Pearson correlation coefficient is 0.96464, the R-square, which is also known as the coefficient of determination (COD), is 0.93054, and the adjusted R-square is 0.92519. Thus, the fitting result is good. Figure 5 shows the correlation between the I···N interatomic distances (*d*_I···N_) of halogen bonds and corresponding Se···N interatomic distances (*d*_Se···N_) of chalcogen bonds in the fourteen tetramers. The *d*_Se···N_ increases as *d*_I···N_ increases, and the two exhibit a clear positive linear correlation. The values of the three different correlation coefficients are all close to 1, indicating a very strong correlation between *d*_Se···N_ and *d*_I···N_.

Performing the energy decomposition analyses for the total interaction energies of the [Se–N]_2_ supramolecular synthons helps us to understand the nature of the noncovalent bonds in these synthons. Currently, more than fifteen different energy decomposition analysis methods have been developed and applied [39]. In this study, we use the generalized Kohn–Sham energy decomposition analysis (GKS-EDA) method developed by Su et al. to perform energy decomposition analyses of the total interaction energies [40,41]. In the GKS-EDA scheme, the total interaction energy is divided into six energy components: electrostatic (∆*E*^ele^), exchange (∆*E*^ex^), repulsion (∆*E*^rep^), polarization (∆*E*^pol^), correlation (∆*E*^corr^), and dispersion (∆*E*^disp^) terms. Further details for the GKS-EDA method can be found in references [40,41]. Table 2 lists the results of GKS-EDA for the total interaction energies of BSeD···BSeD in different tetramers. The ∆*E*^exrep^ in Table 2 is the sum of ∆*E*^ex^ and ∆*E*^rep^. The ∆*E*^exrep^ term is repulsive, while the other four terms in Table 2 are attractive. The contribution of the ∆*E*^ele^ term to the total attractive interaction energy is much greater than the contributions of the other attractive terms to the total attractive interaction energy. In some studies, the ∆*E*^corr^ (short-range dispersion) term and the ∆*E*^disp^ (long-range dispersion) term are often combined into a single term representing the total dispersion term [42]. Even so, the contribution of the ∆*E*^ele^ term to the total attractive interaction energy is still much greater than the contribution of the total dispersion term to the total attractive interaction energy. These computational results show that the electrostatic energy plays a dominant role in stabilizing [Se–N]_2_ supramolecular synthons. It is reasonable to analyze the effect of halogen bonds on [Se–N]_2_ supramolecular synthons based on the changes in electrostatic potential. This also aligns with our chemical intuition.

As mentioned earlier, an increase in the electrostatic potential of the Se atom’s σ-hole region enhances the strength of the Se···N chalcogen bond, while an increase in the electrostatic potential of the N atom’s lone pair electron region weakens the strength of the Se···N chalcogen bond. Here, the result that the [Se–N]_2_ supramolecular synthon becomes stronger under the effect of the I···N halogen bond suggests that the change in the electrostatic potential of the Se atom’s σ-hole region plays a dominant role, while the change in the electrostatic potential of the N atom’s lone pair electron region plays a secondary role.

### 2.2. The Effect of Halogen Bonds on [Se–N]_2_ Supramolecular Synthons in the Crystalline Phase

In order to study the effect of the I···N halogen bonds on [Se–N]_2_ supramolecular synthons in the crystalline phase, we first conducted a search for the structural motif –I···[=N–Se–N=]_2_···I– in the Cambridge Structural Database. The results revealed that only five crystal structures contain this structural motif. The refcodes of the five cocrystals are GAJQUG, GAJRER, GAJQIU, MAVHUQ, and MAVJIG, respectively [14,19]. In the crystal structures of MAVHUQ and MAVJIG, various noncovalent interactions are intertwined, making it difficult to accurately calculate the interaction energies of the I···N halogen bonds and the [Se–N]_2_ supramolecular synthons. Ultimately, only three cocrystals—GAJQUG, GAJRER, and GAJQIU—could be used for analysis, resulting in a small sample size. Considering that the structural motif –I···[=N–Se–N=]_2_···I– is relatively strong, cocrystals assembled through this motif should be easier to synthesize. With BSeD and perfluoroiodobenzenes as the two components, we have also successfully synthesized three additional cocrystals containing this structural motif.

**Figure 6:**
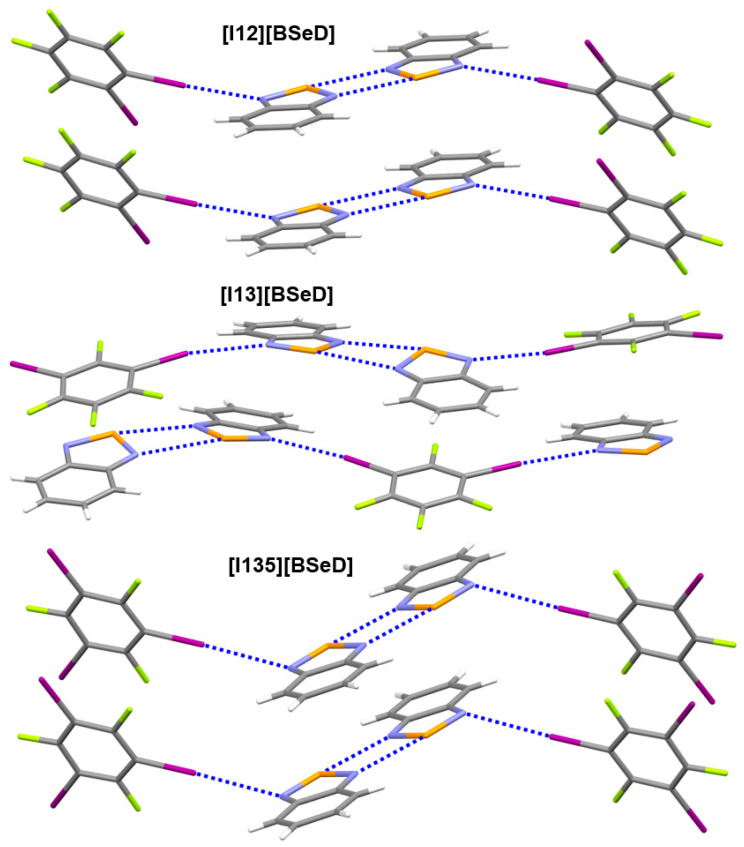
The strong noncovalent interactions in the crystal structures of [I12][BSeD], [I13][BSeD], and [I135][BSeD]. Color code: H, white; C, gray; N, blue; F, yellow-green; Se, orange; I, purple.

Table 3 lists the crystal and X-ray structure refinement data for the three cocrystals [I12][BSeD], [I13][BSeD], and [I135][BSeD]. Here, I12 represents the 1,2-diiodotetrafluorobenzene molecule, I13 represents the 1,3-diiodotetrafluorobenzene molecule, and I135 represents the 1,3,5-trifluoro-2,4,6-triiodobenzene molecule. The cocrystal [I13][BSeD] is of the orthorhombic crystal system, while the cocrystals [I12][BSeD] and [I135][BSeD] belong to the same crystal system, which is monoclinic. Figure 6 shows the strong noncovalent interactions in the crystal structures of [I12][BSeD], [I13][BSeD], and [I135][BSeD]. Other weak noncovalent interactions in the three crystal structures can be viewed from the crystallographic information files (CIFs) available as Supplementary Materials. The motif –I···[=N–Se–N=]_2_···I– exists in each of the three cocrystals. Since the crystal systems of the cocrystals [I12][BSeD] and [I135][BSeD] are the same, the noncovalent interactions in their crystal structures are also very similar. In addition to the I···N halogen bonds and Se···N chalcogen bonds, the crystal structure of [I12][BSeD] also contains π···π stacking interactions between I12 molecules and π···π stacking interactions between BSeD molecules, and the crystal structure of [I135][BSeD] also includes π···π stacking interactions between I135 molecules and π···π stacking interactions between BSeD molecules. In the crystal structure of [I13][BSeD], there are no π···π stacking interactions between I13 molecules, and instead, there are π···π stacking interactions between [I13] and [BSeD] molecules. As shown in Figure 6, in the crystal structures of [I12][BSeD] and [I135][BSeD], the two BSeD molecules forming the [Se–N]_2_ supramolecular synthon are almost coplanar. However, in the crystal structure of [I13][BSeD], the two BSeD molecules forming the [Se–N]_2_ supramolecular synthon exhibit a certain torsion angle and are not coplanar. The comparison of noncovalent interactions in these three crystals shows that the intermolecular π···π stacking interactions determine whether the two BSeD molecules that form the [Se–N]_2_ supramolecular synthon lie in the same plane or not.

**Figure 7:**
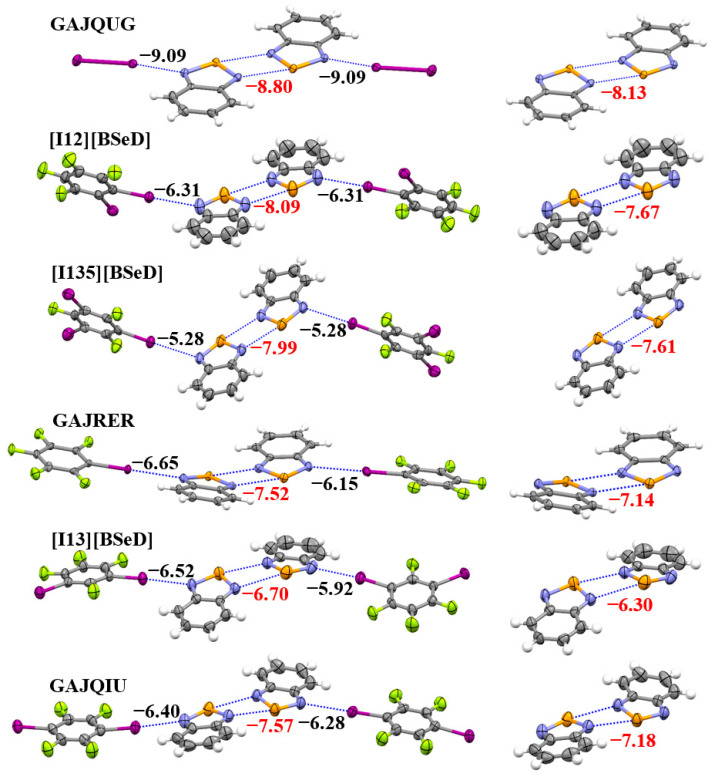
The PBE0-D3/def2-TZVPP interaction energies (kcal/mol) of the I···N halogen bonds and [Se–N]_2_ supramolecular synthons in the crystal structures of six studied cocrystals. Color code: H, white; C, gray; N, blue; F, yellow-green; Se, orange; I, purple.

Figure 7 shows the tetramers (left) and dimers (right) in the crystal structures of GAJQUG, [I12][BSeD], [I135][BSeD], GAJRER, [I13][BSeD], and GAJQIU, along with the interaction energies of the I···N halogen bonds and [Se–N]_2_ supramolecular synthons calculated at the PBE0-D3/def2-TZVPP level of theory. Although the introduction of I···N halogen bonds enhances the [Se–N]_2_ supramolecular synthons, stronger I···N halogen bonds do not lead to stronger [Se–N]_2_ supramolecular synthons. For example, the I···N halogen bonds in the crystal structure of [I135][BSeD] are weaker than the I···N halogen bonds in the crystal structures of GAJRER, [I13][BSeD] and GAJQIU, while the [Se–N]_2_ supramolecular synthon in the [I135][BSeD] crystal structure is stronger than the [Se–N]_2_ supramolecular synthons in the crystal structures of GAJRER, [I13][BSeD] and GAJQIU. However, such a conclusion requires further analysis. As can be seen from Figure 7, the two I···N halogen bonds in each of the crystal structures of GAJQUG, [I12][BSeD] and [I135][BSeD] are symmetric, with the two BSeD molecules forming the [Se–N]_2_ supramolecular synthon almost lying in the same plane. In contrast, the two I···N halogen bonds in each of the crystal structures of GAJRER, [I13][BSeD] and GAJQIU are asymmetric, and the two BSeD molecules forming the [Se–N]_2_ supramolecular synthon are not in the same plane. This difference is clearly due to the effect of other noncovalent interactions in the crystal structures. Only the comparison of noncovalent bonds in the crystal structures of the three cocrystals, GAJQUG, [I12][BSeD], and [I135][BSeD], is meaningful. Although the sample size is small, with only three crystals, we can still draw two conclusions: (1) the introduction of the I···N halogen bonds strengthens the [Se–N]_2_ supramolecular synthon, and (2) as the I···N halogen bonds become stronger, the strength of the [Se–N]_2_ supramolecular synthon also increases, showing a proportional relationship. This is consistent with the results in the gas phase.

In this study, we exclusively focused on tetrameric systems without addressing the potential influences from other noncovalent interactions. As demonstrated by Figures S1 and S2 in the Supplementary Materials, any hypothetical enhancement of halogen bonds through additional noncovalent interactions would proportionally strengthen the [Se–N]_2_ supramolecular synthons and vice versa. This reciprocity confirms that omitting such interactions does not compromise the validity of our conclusions.

### 2.3. Expansion of the [Se–N]_2_ Supramolecular Synthon

In the previous text, we selected BSeD as a representative molecule of selenadiazole to study the effect of I···N halogen bonds on [Se–N]_2_ supramolecular synthons. If BSeD were replaced with other selenadiazole molecules, the conclusions obtained should, in principle, remain the same. To verify this hypothesis, we tried to synthesize the cocrystals formed by F-BSeD with I_2_, I12, I13, 1,4-diiodotetrafluorobenzene (I14) and I135, respectively. Finally, only two cocrystals, [I14][F-BSeD] and [I135][F-BSeD], were successfully synthesized and resolved. Similarly, the effect of the I···N halogen bonds on the [Se–N]_2_ supramolecular synthons formed by the F-BSeD molecule was studied.

**Figure 8:**
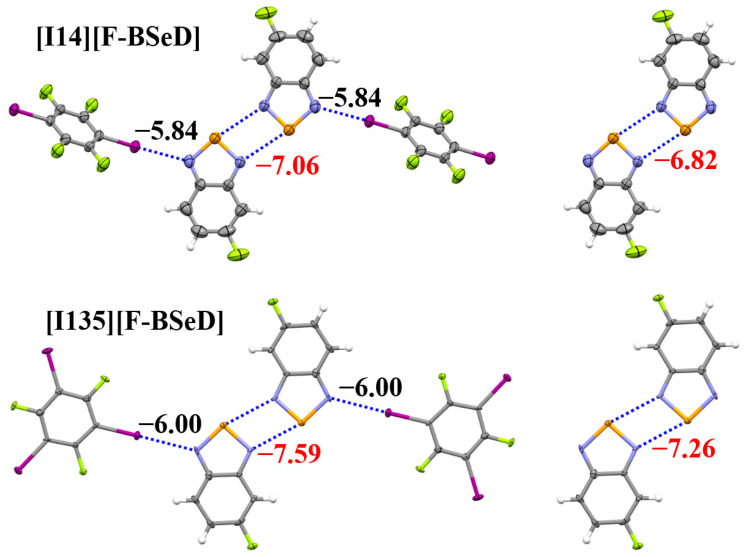
The PBE0-D3/def2-TZVPP interaction energies (kcal/mol) of the I···N halogen bonds and [Se–N]_2_ supramolecular synthons in the crystal structures of [I14][F-BSeD] and [I135][F-BSeD]. Color code: H, white; C, gray; N, blue; F, yellow-green; Se, orange; I, purple.

Table 4 summarizes the crystal and X-ray structure refinement data for the two cocrystals [I14][F-BSeD] and [I135][F-BSeD]. The CIF files of [I14][F-BSeD] and [I135][F-BSeD] have been given in the Supplementary Materials. The crystal structures of [I14][F-BSeD] and [I135][F-BSeD] both belong to the monoclinic system. The noncovalent interactions in the crystal structures of [I14][F-BSeD] and [I135][F-BSeD] are also very similar. In addition to the I···N halogen bonds and Se···N chalcogen bonds that we focused on, there are also π···π stacking interactions between the F-BSeD molecules and π···π stacking interactions between the F-BSeD and I14/I135 molecules. Figure 8 shows the tetramers studied in the crystal structures of [I14][F-BSeD] and [I135][F-BSeD], along with the corresponding dimers for comparison. Both the tetramer and dimer are symmetrical structures. Meanwhile, the two F-BSeD molecules that form the [Se–N]_2_ supramolecular synthons are approximately in the same plane. The interaction energies of the I···N halogen bonds and [Se–N]_2_ supramolecular synthons are also shown in Figure 8. The interaction energy from the dimer to the tetramer indicates that the formation of I···N halogen bonds strengthens the [Se–N]_2_ supramolecular synthons. Additionally, by comparing the interaction energies of the tetramers in the crystal structures of [I14][F-BSeD] and [I135][F-BSeD], we observe that as the I···N halogen bonds strengthen and the [Se–N]_2_ supramolecular synthons also become stronger, with a proportional relationship between the I···N halogen bonds and [Se–N]_2_ supramolecular synthons. These results are consistent with the corresponding findings for the [Se–N]_2_ supramolecular synthons formed by BSeD, confirming our hypothesis. Furthermore, it is believed that the conclusions drawn in this paper are also applicable to the [Se–N]_2_ supramolecular synthons formed by other selenadiazoles or their derivatives.

In the crystal structure of [I14][F-BSeD], the distances of *d*_I···N_ and *d*_Se···N_ are 2.987 Å and 3.069 Å, respectively. In the crystal structure of [I135][F-BSeD], these distances measure *d*_I···N_ = 3.012 Å and *d*_Se···N_ = 2.848 Å. These results contradict the proportional relationship observed between *d*_I···N_ and *d*_Se···N_ in the gas phase. The strength of a halogen bond depends not only on its interatomic distance but also on its angle and dihedral angle. In gas-phase calculations, tetramers predominantly adopt planar configurations where the angles and dihedral angles of halogen bonds remain essentially constant, resulting in strong linear correlations between *d*_I···N_ and *d*_Se···N_. However, crystalline environments introduce significantly greater structural complexity, where the linear correlation between *d*_I···N_ and *d*_Se···N_ may no longer hold due to the effects of other noncovalent interactions. In contrast to the crystal structure of [I135][F-BSeD], the C–I bond of the I14 molecule in the crystal structure of [I14][F-BSeD] deviates from coplanarity with the F-BSeD molecule, exhibiting an angular displacement of approximately 24°. This geometric deviation results in a shorter *d*_I···N_ distance compared to that in the [I135][F-BSeD] system, yet paradoxically weakens the corresponding I···N halogen bond strength. This example clearly demonstrates that changes in the relative positions of molecules can reverse the relationship between noncovalent bond strength and noncovalent bond length. Consequently, analyses of interaction energies rather than interatomic distances are prioritized in crystal structure discussions involving these systems.

## 3. Materials and Methods

### 3.1. Computational Details

**Figure 9:**
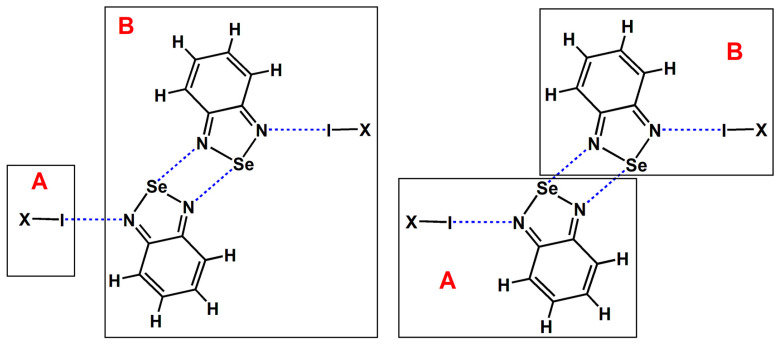
The division of fragment A and fragment B for the calculations of the I···N halogen bonds and [Se–N]_2_ supramolecular synthons in the tetramers.

In the gas phase, the geometries of the tetramers were fully optimized at the PBE0-D3/def2-TZVPP level of theory [35,36,37]. In the crystalline phase, the geometries of the tetramers were not optimized and taken directly from the crystal structures. For comparison, the PBE0-D3/def2-TZVPP calculations of the dimers used their corresponding geometries in the tetramers. The electrostatic potentials and interaction energies were also calculated at the PBE0-D3/def2-TZVPP theory level. Our previous studies have confirmed the reliability of the PBE0-D3/def2-TZVPP calculations for accurately describing the noncovalent interactions [43,44,45]. In fact, the PBE0-D3/def2-TZVPP calculations were often used in the study of noncovalent interactions in crystal structures [46,47,48,49,50,51]. The electrostatic potential maps were plotted on the 0.001 au electron density isosurfaces. All the interaction energies have been corrected for basis set superposition error by using the counterpoise scheme of Boys and Bernardi [52]. The interaction energies (∆*E*) were calculated with the supermolecule method. The calculation formula is as follows:

∆*E* = *E*^AB^ − *E*^A^ − *E*^B^

where *E*^AB^ is the energy of the complex AB, *E*^A^ is the energy of fragment A, and *E*^B^ is the energy of fragment B. Therefore, in order to calculate the interaction energies of the I···N halogen bonds and [Se–N]_2_ supramolecular synthons in the tetramers, each of the tetramers was separated into its fragment A and fragment B subunits (see Figure 9). Note that we only illustrate the division of the tetramer formed by BSeD in Figure 9. The division method for the tetramer formed by F-BSeD is the same.

The GKS-EDA calculations were carried out with the Xiamen Atomistic Computing Suite [40,41]. The other calculations were performed with the Gaussian 16 suite of programs [53].

### 3.2. Syntheses of Cocrystals

I12 (purity: ≥98%), I13 (purity: ≥98%), I14 (purity: ≥98%), and I135 (purity: ≥98%) were purchased from J&K Scientific Ltd., Beijing, China. BSeD (purity: ≥98%) and F-BSeD (purity: ≥98%) were purchased from Alfa Chemical Co., Ltd., Zhengzhou, China. The solvents (analytical reagent grade) were purchased from local suppliers. All reagents and solvents were used without further purification. The synthesis steps for the five cocrystals [I12][BSeD], [I13][BSeD], [I135][BSeD], [I14][F-BSeD], and [I135][F-BSeD] are the same. We weighed 0.1 mmol of the halogen-bond donor (I12, I13, I14, or I135) and 0.1 mmol of the corresponding halogen-bond acceptor (BSeD or F-BSeD) and dissolved them in 10 mL of chloroform solvent. The solution was gently stirred for 30 min and then filtered. The filtrate was allowed to slowly evaporate at room temperature, and after 2–3 days, single crystals suitable for X-ray diffraction measurements were obtained. In fact, we also attempted to synthesize other cocrystals of the same series. Since the attempts were unsuccessful, the synthesis details will not be reiterated here.

### 3.3. X-Ray Structure Determinations

Single-crystal X-ray diffraction data were collected on the Oxford Diffraction SuperNova area-detector diffractometer equipped with the Mo-Kα X-ray source (λ = 0.71073 Å). The cell refinements and data reduction were carried out by using the CrysAlisPro 1.171.40.53 software package [54]. The crystal structure was solved with the ShelXT and ShelXL programs [55,56]. The H atoms in all structures were refined at idealized positions riding on the C atoms, with isotropic displacement parameters *U*_iso_(H) = 1.2*U*_eq_(C) and *d*(C–H) = 0.93 Å. The CIF files of the five cocrystals (CCDC deposition numbers: 2239822-2239824, 2417680, 2417681) can be obtained free of charge via https://www.ccdc.cam.ac.uk/structures (accessed on 17 January 2025) At the same time, the CIF files of the five cocrystals were also provided as electronic Supplementary Materials. The checkCIF files for the five cocrystal structures can be found in the Supplementary Materials.

## 4. Conclusions

The [Se–N]_2_ supramolecular synthon is one of the most important synthons formed by chalcogen bonds in the field of supramolecular chemistry. In this study, the effect of the I···N halogen bonds on [Se–N]_2_ supramolecular synthons has been investigated in detail by means of a combined theoretical calculation and single-crystal X-ray crystallographic experiment approach.

The results of the gas-phase calculations show that the formation of I···N halogen bonds significantly strengthens [Se–N]_2_ supramolecular synthons. At the same time, as the I···N halogen bonds strengthen, the [Se–N]_2_ supramolecular synthons also increase, and there is a very good correlation between the I···N halogen bonds and [Se–N]_2_ supramolecular synthons. Due to the effect of other noncovalent interactions, the relationship between the I···N halogen bonds and [Se–N]_2_ supramolecular synthons in the crystal structure is relatively more complex. However, if the effect of other noncovalent interactions is relatively small, the strength of the halogen bond remains directly proportional to the strength of the [Se–N]_2_ supramolecular synthons.

Now, we can provide a clear answer to the question raised in the introduction section. That is, the positive shift in the electrostatic potential at the region of the Se atom’s σ-hole, which leads to the enhancement of the [Se–N]_2_ supramolecular synthon, plays a dominant role, while the positive shift at the region of the lone pair electrons of the N atom, which weakens the [Se–N]_2_ supramolecular synthon, plays a secondary role. At the same time, our study also finds that, during the process in which I···N halogen bonds enhance the [Se–N]_2_ supramolecular synthons, polarization energy and dispersion energy play secondary roles, with electrostatic energy dominating. This is consistent with our chemical intuition.

We also extended the [Se–N]_2_ supramolecular synthons formed by BSeD to those formed by F-BSeD and found that the positive correlation between I···N halogen bonds and [Se–N]_2_ supramolecular synthons still holds. The two unbound N atoms in [Se–N]_2_ supramolecular synthons can also form two hydrogen bonds [14]. We believe that the conclusions of this study would still hold if the I···N halogen bonds in this work were replaced with other types of noncovalent interactions, such as hydrogen bonds.

## Figures and Tables

**Table 1:** The interatomic distances in the tetramers (*d*_I···N_ and *d*_Se···N_). Total interaction energies of the halogen bonds in the tetramers (${∆E}_{Tot}^{XB}$(T)), total interaction energies of the chalcogen bonds in the tetramers and BSeD···BSeD dimers (${∆E}_{Tot}^{ChB}$(T) and ${∆E}_{Tot}^{ChB}$(D)), and differences between ${∆E}_{Tot}^{ChB}$(T) and ${∆E}_{Tot}^{ChB}$(D) calculated at the PBE0-D3/def2-TZVPP level of theory. The interatomic distances are in Å, and the energies are in kcal/mol.

Tetramer	*d*_I···N_	*d*_Se···N_	${∆E}_{Tot}^{XB}$(T)	${∆E}_{Tot}^{ChB}$(T)	${∆E}_{Tot}^{ChB}$(D)	${∆E}_{Tot}^{ChB}$$(T)-{∆E}_{Tot}^{ChB}$(D)
FI···BSeD···BSeD···FI	2.401	2.795	−19.44	−9.23	−8.51	−0.72
ClI···BSeD···BSeD···ClI	2.516	2.814	−14.05	−8.98	−8.35	−0.63
BrI···BSeD···BSeD···BrI	2.563	2.821	−12.35	−8.90	−8.29	−0.61
I_2_···BSeD···BSeD···I_2_	2.690	2.837	−9.32	−8.69	−8.12	−0.57
CF_3_I···BSeD···BSeD···CF_3_I	2.957	2.868	−6.06	−8.23	−7.81	−0.42
CCl_3_I···BSeD···BSeD···CCl_3_I	2.847	2.858	−7.13	−8.39	−7.91	−0.48
CBr_3_I···BSeD···BSeD···CBr_3_I	2.823	2.855	−7.49	−8.45	−7.93	−0.52
CI_4_···BSeD···BSeD···CI_4_	2.825	2.855	−7.39	−8.47	−7.93	−0.54
HCCI···BSeD···BSeD···HCCI	2.930	2.867	−6.62	−8.24	−7.81	−0.43
FCCI···BSeD···BSeD···FCCI	2.904	2.866	−6.92	−8.27	−7.83	−0.44
NCI···BSeD···BSeD···NCI	2.819	2.853	−8.91	−8.37	−7.94	−0.43
C_6_F_3_I_3_···BSeD···BSeD···C_6_F_3_I_3_	2.935	2.866	−6.34	−8.28	−7.82	−0.46
C_6_F_4_I_2_···BSeD···BSeD···C_6_F_4_I_2_	2.928	2.865	−6.53	−8.30	−7.83	−0.47
C_6_F_5_I···BSeD···BSeD···C_6_F_5_I	2.926	2.865	−6.55	−8.29	−7.83	−0.46

**Table 2:** Energy components of the total interaction energies of BSeD···BSeD in different tetramers calculated with the GKS-EDA method. All energies are in kcal/mol.

Dimer	∆*E*^ele^	∆*E*^exrep^	∆*E*^pol^	∆*E*^corr^	∆*E*^disp^
BSeD···BSeD(FI···BSeD···BSeD···FI)	−21.40	30.89	−9.40	−7.23	−3.38
BSeD···BSeD(ClI···BSeD···BSeD···ClI)	−20.33	31.00	−8.80	−6.90	−3.34
BSeD···BSeD(BrI···BSeD···BSeD···BrI)	−19.95	30.36	−8.60	−6.78	−3.32
BSeD···BSeD(I_2_···BSeD···BSeD···I_2_)	−19.06	28.84	−8.12	−6.51	−3.29
BSeD···BSeD(CF_3_I···BSeD···BSeD···CF_3_I)	−17.44	26.13	−7.26	−6.04	−3.21
BSeD···BSeD(CCl_3_I···BSeD···BSeD···CCl_3_I)	−17.95	26.99	−7.52	−6.19	−3.24
BSeD···BSeD(CBr_3_I···BSeD···BSeD···CBr_3_I)	−18.10	27.23	−7.60	−6.23	−3.24
BSeD···BSeD(CI_4_···BSeD···BSeD···CI_4_)	−18.09	27.21	−7.60	−6.22	−3.24
BSeD···BSeD(HCCI···BSeD···BSeD···HCCI)	−17.49	26.22	−7.29	−6.04	−3.22
BSeD···BSeD(FCCI···BSeD···BSeD···FCCI)	−17.55	26.31	−7.31	−6.06	−3.22
BSeD···BSeD(NCI···BSeD···BSeD···NCI)	−18.21	27.44	−7.66	−6.27	−3.25
BSeD···BSeD(C_6_F_3_I_3_···BSeD···BSeD···C_6_F_3_I_3_)	−17.53	26.27	−7.31	−6.04	−3.22
BSeD···BSeD(C_6_F_4_I_2_···BSeD···BSeD···C_6_F_4_I_2_)	−17.58	26.36	−7.33	−6.06	−3.22
BSeD···BSeD(C_6_F_5_I···BSeD···BSeD···C_6_F_5_I)	−17.59	26.39	−7.34	−6.08	−3.22

**Table 3:** Crystal and X-ray structure refinement data for the three cocrystals [I12][BSeD], [I13][BSeD], and [I135][BSeD].

	[I12][BSeD]	[I13][BSeD]	[I135][BSeD]
CCDC deposition number	2239822	2239823	2239824
Empirical formula	C_12_H_4_F_4_I_2_N_2_Se	C_18_H_8_F_4_I_2_N_4_Se_2_	C_12_H_4_F_3_I_3_N_2_Se
Formula weight	584.93	768.00	692.83
Temperature/K	293.00(2)	290.00(10)	293.00(2)
Crystal system	monoclinic	orthorhombic	monoclinic
Space group	*P*2_1_/*n*	*Pbca*	*P*2_1_/*c*
*a*/Å	15.0997(6)	14.8028(4)	4.4302(2)
*b*/Å	4.2279(2)	15.5137(4)	29.9768(14)
*c*/Å	23.6753(9)	37.2860(14)	12.2962(7)
*α/°*	90	90	90
*β/°*	90.124(4)	90	91.505(5)
*γ/°*	90	90	90
Volume/Å^3^	1511.43(11)	8562.6(5)	1632.41(14)
*Z*	4	16	4
*ρ*_calc_/g·cm^−3^	2.571	2.383	2.819
Color	colorless	colorless	colorless
Crystal size/mm^3^	0.29 × 0.28 × 0.17	0.2 × 0.17 × 0.15	0.22 × 0.18 × 0.15
Reflections collected	16,599	57,076	19,226
Independent reflections	3453	9660	3668
*R*_int_	0.0371	0.0693	0.0547
Number of refined parameters	190	541	190
Goodness-of-fit on *F*^2^	1.122	1.094	1.308
Final *R*_1_ index [I ≥ 2*σ*(I)]	0.0333	0.060	0.0770
Final *wR*_2_ index [I ≥ 2*σ*(I)]	0.0623	0.0756	0.1240
Final *R*_1_ index [all data]	0.0424	0.1217	0.0918
Final *wR*_2_ index [all data]	0.0657	0.0880	0.1286

**Table 4:** Crystal and X-ray structure refinement data for the two cocrystals [I14][F-BSeD] and [I135][F-BSeD].

	[I14][F-BSeD]	[I135][F-BSeD]
CCDC deposition number	2417680	2417681
Empirical formula	C_18_H_6_F_6_I_2_N_4_Se_2_	C_18_H_6_F_5_I_3_N_4_Se_2_
Formula weight	803.99	911.89
Temperature/K	293.00(2)	100.00(10)
Crystal system	monoclinic	monoclinic
Space group	*P*2_1_/*n*	*C*2/*c*
*a*/Å	13.0258(6)	14.3599(6)
*b*/Å	6.3844(3)	9.2659(4)
*c*/Å	13.4476(6)	17.4527(9)
*α/°*	90	90
*β/°*	104.852(5)	101.082(4)
*γ/°*	90	90
Volume/Å^3^	1080.97(9)	2278.91(18)
*Z*	2	4
*ρ*_calc_/g·cm^−3^	2.470	2.658
Color	colorless	colorless
Crystal size/mm^3^	0.33 × 0.25 × 0.18	0.13 × 0.12 × 0.10
Reflections collected	13343	2009
Independent reflections	2678	2009
*R*_int_	0.0627	0.0334
Number of refined parameters	145	142
Goodness-of-fit on *F*^2^	1.036	1.113
Final *R*_1_ index [I ≥ 2*σ*(I)]	0.0349	0.0674
Final *wR*_2_ index [I ≥ 2*σ*(I)]	0.0555	0.1818
Final *R*_1_ index [all data]	0.0579	0.0716
Final *wR*_2_ index [all data]	0.0626	0.1857

## Data Availability

Data are available on request from the authors.

## Supplementary Materials

The following supporting information can be downloaded at: https://www.mdpi.com/article/10.3390/ijms26052324/s1.
