# Supplementary material for: Chalcogen-Bonded [Se–N]2 Cyclic Supramolecular Synthons Enhanced by Halogen Bonds: Studies in the Gas Phase and Crystalline Phase
Source: Int J Mol Sci. 2025 Mar 5;26(5):2324. doi: 10.3390/ijms26052324 (PMC11900184; doi:10.3390/ijms26052324)
Supplement: Supplementary file 1 [file ijms-26-02324-s001.zip › Supplementary Materials/File S1.pdf]

Electronic supplementary materials

Chalcogen-Bonded $[Se–N]_2$ Cyclic Supramolecular Synthons Enhanced by Halogen Bonds: Studies in the Gas Phase and Crystalline Phase

Shaobin Miao, Xiaotian Sun, Yu Zhang and Weizhou Wang *

College of Chemistry and Chemical Engineering, and Henan Key Laboratory of Function-Oriented Porous Materials, Luoyang Normal University, Luoyang 471934, China; miaoshaobin@126.com (S.M.); sxt@lynu.edu.cn (X.S.); yzhpaper@yahoo.com (Y.Z.)

* Correspondence: wzw@lynu.edu.cn; Tel.: +86-379-686-18320

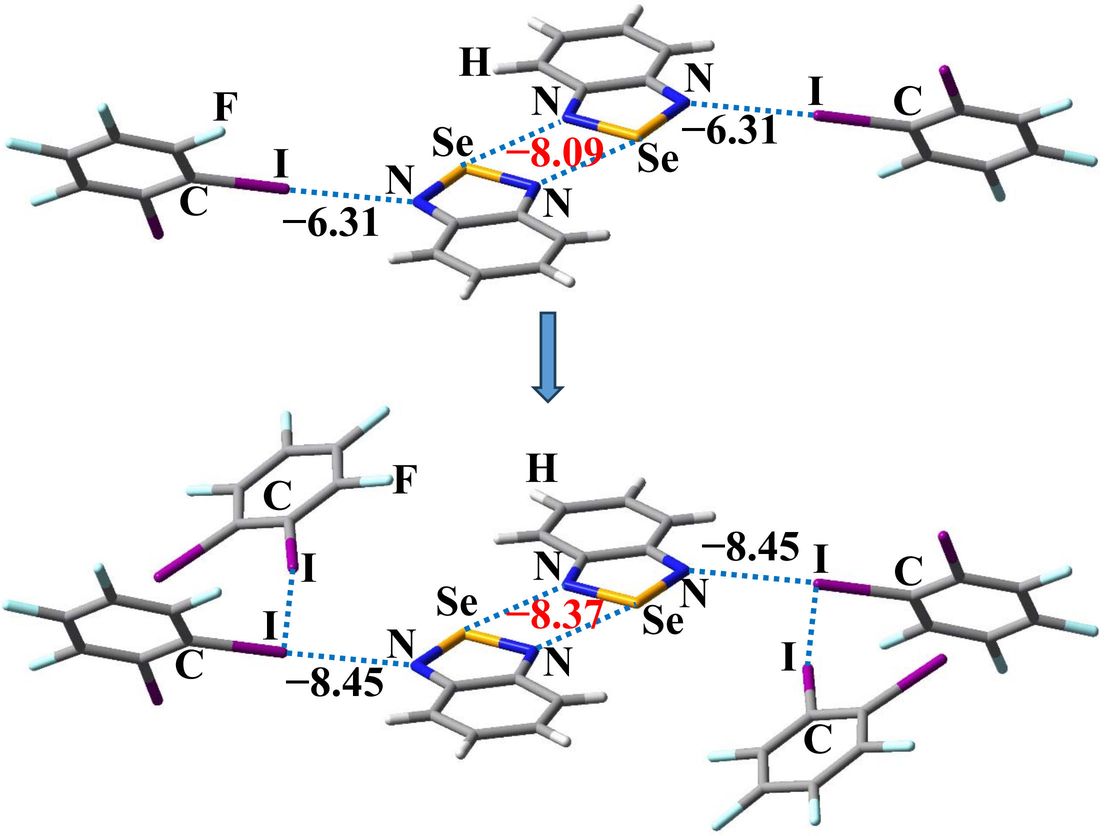

Figure S1. The PBE0-D3/def2-TZVPP interaction energies (kcal/mol) of the I···N halogen bonds and [Se–N]$_2$ supramolecular synthons in the crystal structures of [I12][BSeD]. The upper panel of the figure shows the tetramer without considering the effects of the I···I halogen bonds, while the lower panel depicts the hexamer with incorporation of the effects of the I···I halogen bonds. The geometries of the tetramer and hexamer were not optimized and were directly extracted from the crystal structure of [I12][BSeD]. Color code: H, white; C, gray; N, blue; F, cyan; Se, orange; I, purple. **When the I···N halogen bonds are enhanced by the cooperative interactions of the I···I halogen bonds, the [Se–N]$_2$ supramolecular synthons are concurrently strengthened. Thus, inclusion or exclusion of the I···I halogen bonds does not affect the conclusions of this study.**

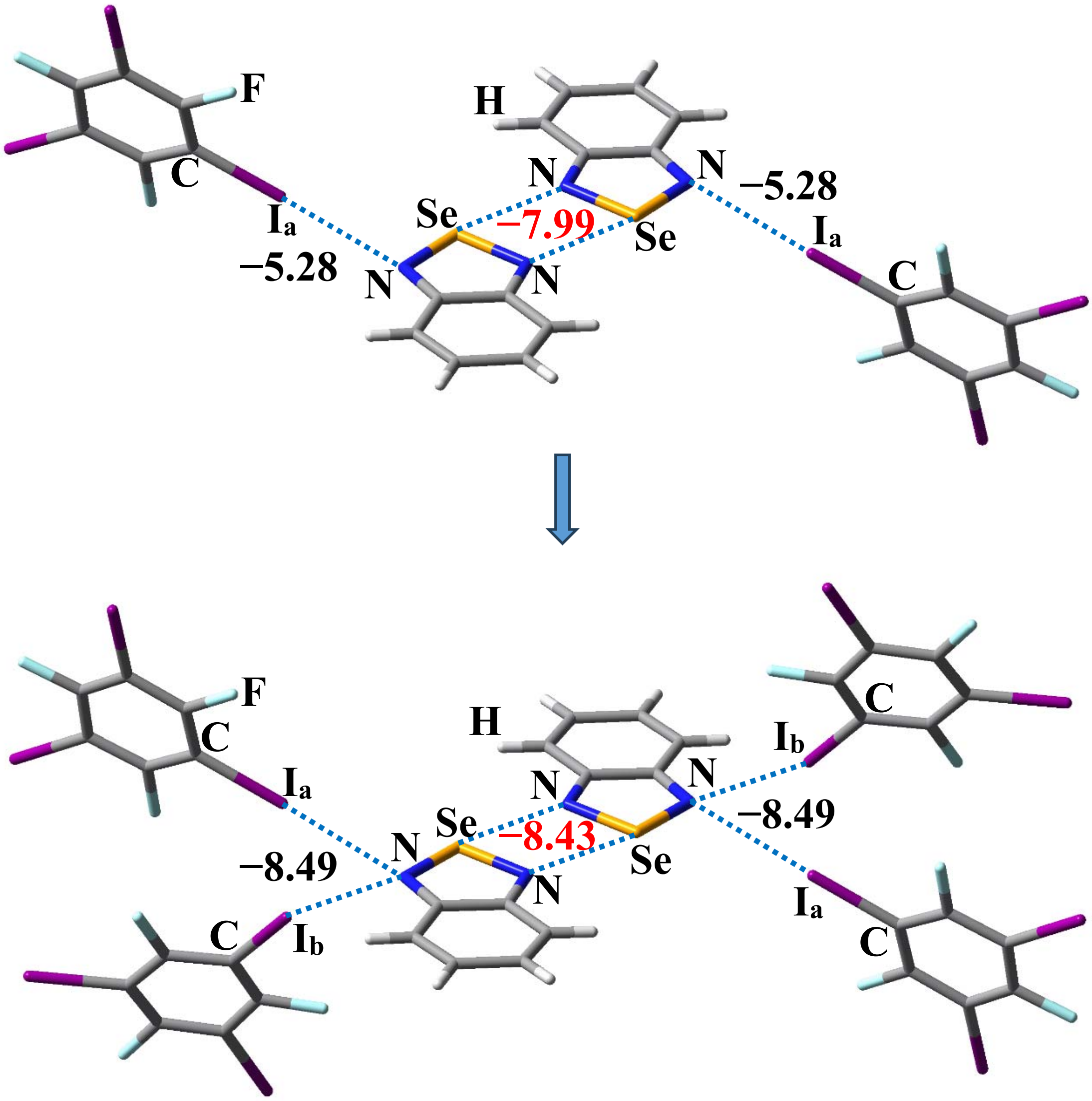

Figure S2. The PBE0-D3/def2-TZVPP interaction energies (kcal/mol) of the bifurcated (I_a,I_b)···N halogen bonds and $[Se–N]_2$ supramolecular synthons in the crystal structures of [I135][BSeD]. The upper panel of the figure shows the tetramer without considering the effects of the I_b···N halogen bonds, while the lower panel depicts the hexamer with incorporation of the effects of the I_b···N halogen bonds. The geometries of the tetramer and hexamer were not optimized and were directly extracted from the crystal structure of [I135][BSeD]. Color code: H, white; C, gray; N, blue; F, cyan; Se, orange; I, purple. **The bifurcated (I_a,I_b)···N halogen bonds are stronger than the I_a···N halogen bonds, which leads to that the $[Se–N]_2$ supramolecular synthons are concurrently strengthened. Thus, inclusion or exclusion of the I_b···N halogen bonds does not affect the conclusions of this study.**
