# Supplementary material for: Chalcogen-Bonded [Se–N]2 Cyclic Supramolecular Synthons Enhanced by Halogen Bonds: Studies in the Gas Phase and Crystalline Phase
Source: Int J Mol Sci. 2025 Mar 5;26(5):2324. doi: 10.3390/ijms26052324 (PMC11900184; doi:10.3390/ijms26052324)

checkCIF/PLATON report

Structure factors have been supplied for datablock(s) 2239822

THIS REPORT IS FOR GUIDANCE ONLY. IF USED AS PART OF A REVIEW PROCEDURE FOR PUBLICATION, IT SHOULD NOT REPLACE THE EXPERTISE OF AN EXPERIENCED CRYSTALLOGRAPHIC REFEREE.

No syntax errors found. CIF dictionary Interpreting this report

Datablock: 2239822

Bond precision:	C-C = 0.0070 A		Wavelength=0.71073
Cell:	a=15.0997(6)	b=4.2279(2)	c=23.6753(9)
	alpha=90	beta=90.124(4)	gamma=90
Temperature:	293 K		

	Calculated	Reported
Volume	1511.43(11)	1511.43(11)
Space group	P 21/n	P 1 21/n 1
Hall group	-P 2yn	-P 2yn
Moiety formula	C6 F4 I2, C6 H4 N2 Se	C6 F4 I2, C6 H4 N2 Se
Sum formula	C12 H4 F4 I2 N2 Se	C12 H4 F4 I2 N2 Se
Mr	584.93	584.93
Dx,g cm-3	2.571	2.571
Z	4	4
Mu (mm-1)	6.605	6.605
F000	1064.0	1064.0
F000'	1060.45	
h,k,lmax	20,5,31	20,5,31
Nref	3814	3453
Tmin,Tmax	0.169,0.325	0.309,1.000
Tmin'	0.128	

Correction method= # Reported T Limits: Tmin=0.309 Tmax=1.000
AbsCorr = MULTI-SCAN

Data completeness= 0.905 Theta(max)= 28.436

R(reflections)= 0.0333(2980) wR2(reflections)= 0.0657(3453)

S = 1.122 Npar= 190

The following ALERTS were generated. Each ALERT has the format
test-name_ALERT_alert-type_alert-level.
Click on the hyperlinks for more details of the test.

Alert level C

```
PLAT906_ALERT_3_C Large K Value in the Analysis of Variance ......      4.691 Check
PLAT910_ALERT_3_C Missing # of FCF Reflection(s) Below Theta(Min).          8 Note
                2   0   0,  -1   0   1,    1   0   1,  -2   0   2,    0   0   2,    2   0   2,
               -1   0   3,    1   0   3,
```

Alert level G

```
PLAT012_ALERT_1_G No       _shelx_res_checksum Found in CIF ......     Please Check
PLAT199_ALERT_1_G Reported _cell_measurement_temperature ..... (K)        293 Check
PLAT200_ALERT_1_G Reported   _diffrn_ambient_temperature ..... (K)        293 Check
PLAT431_ALERT_2_G Short Inter HL..A Contact  I2       ..N1       .       2.93 Ang.
                                                     x,y,z  =      1_555 Check
PLAT912_ALERT_4_G Missing # of FCF Reflections Above STh/L=  0.600        353 Note
PLAT941_ALERT_3_G Average HKL Measurement Multiplicity ..........         4.8 Low
PLAT969_ALERT_5_G The 'Henn et al.' R-Factor-gap value ..........        2.43 Note
              Predicted wR2: Based on SigI**2  2.71 or SHELX Weight  6.03
PLAT978_ALERT_2_G Number C-C Bonds with Positive Residual Density.          1 Info
```

0 **ALERT level A** = Most likely a serious problem - resolve or explain
0 **ALERT level B** = A potentially serious problem, consider carefully
2 **ALERT level C** = Check. Ensure it is not caused by an omission or oversight
8 **ALERT level G** = General information/check it is not something unexpected

3 ALERT type 1 CIF construction/syntax error, inconsistent or missing data
2 ALERT type 2 Indicator that the structure model may be wrong or deficient
3 ALERT type 3 Indicator that the structure quality may be low
1 ALERT type 4 Improvement, methodology, query or suggestion
1 ALERT type 5 Informative message, check

It is advisable to attempt to resolve as many as possible of the alerts in all categories. Often the minor alerts point to easily fixed oversights, errors and omissions in your CIF or refinement strategy, so attention to these fine details can be worthwhile. In order to resolve some of the more serious problems it may be necessary to carry out additional measurements or structure refinements. However, the purpose of your study may justify the reported deviations and the more serious of these should normally be commented upon in the discussion or experimental section of a paper or in the "special_details" fields of the CIF. checkCIF was carefully designed to identify outliers and unusual parameters, but every test has its limitations and alerts that are not important in a particular case may appear. Conversely, the absence of alerts does not guarantee there are no aspects of the results needing attention. It is up to the individual to critically assess their own results and, if necessary, seek expert advice.

Publication of your CIF in IUCr journals

A basic structural check has been run on your CIF. These basic checks will be run on all CIFs submitted for publication in IUCr journals (*Acta Crystallographica*, *Journal of Applied Crystallography*, *Journal of Synchrotron Radiation*); however, if you intend to submit to *Acta Crystallographica Section C* or *E* or *IUCrData*, you should make sure that full publication checks are run on the final version of your CIF prior to submission.

Publication of your CIF in other journals

Please refer to the *Notes for Authors* of the relevant journal for any special instructions relating to CIF submission.

PLATON version of 06/01/2024; check.def file version of 05/01/2024

Datablock 2239822 - ellipsoid plot

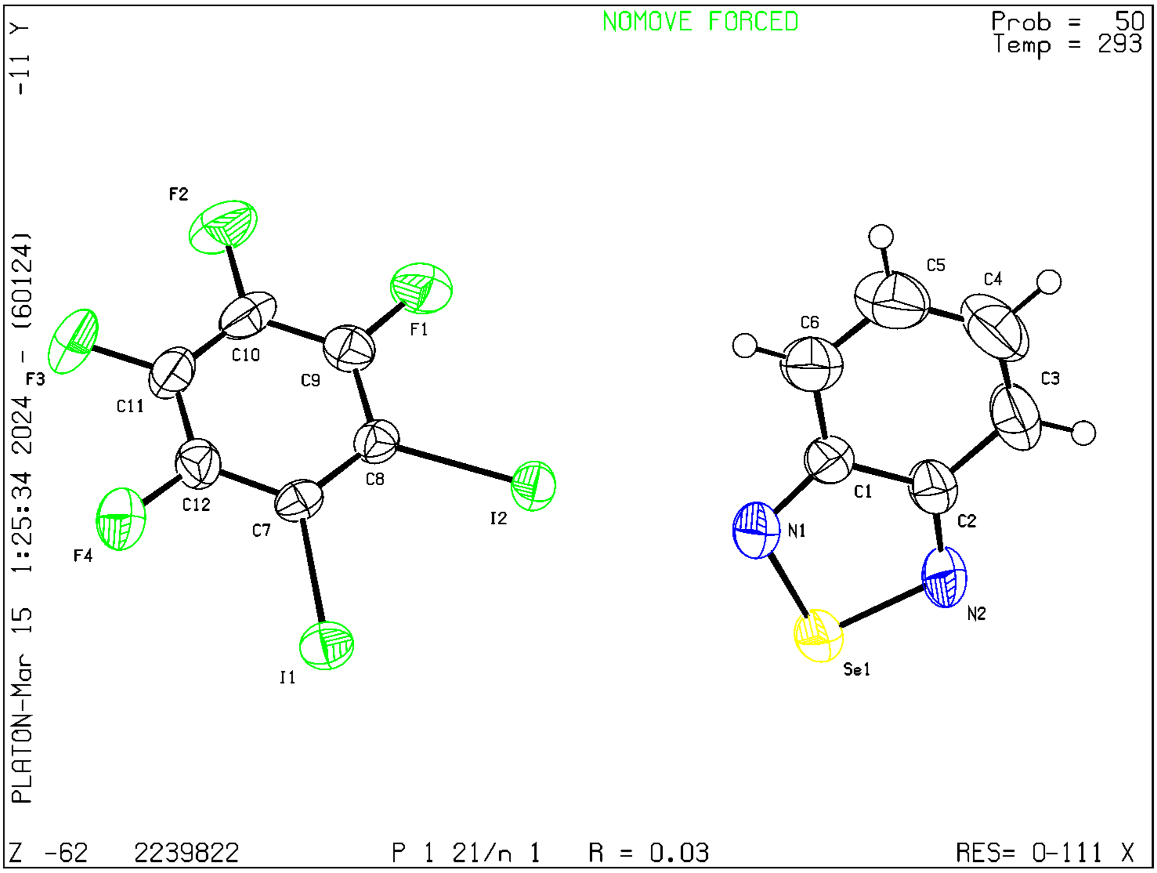

checkCIF/PLATON report

Structure factors have been supplied for datablock(s) 2239823

THIS REPORT IS FOR GUIDANCE ONLY. IF USED AS PART OF A REVIEW PROCEDURE FOR PUBLICATION, IT SHOULD NOT REPLACE THE EXPERTISE OF AN EXPERIENCED CRYSTALLOGRAPHIC REFEREE.

No syntax errors found.   CIF dictionary   Interpreting this report

Datablock: 2239823

Bond precision:	C-C = 0.0101 A		Wavelength=0.71073
Cell:	a=14.8028(4)	b=15.5137(4)	c=37.2860(14)
	alpha=90	beta=90	gamma=90
Temperature:	290 K		

	Calculated	Reported
Volume	8562.6(5)	8562.6(5)
Space group	P b c a	P b c a
Hall group	-P 2ac 2ab	-P 2ac 2ab
Moiety formula	C6 F4 I2, 2(C6 H4 N2 Se)	C6 F4 I2, 2(C6 H4 N2 Se)
Sum formula	C18 H8 F4 I2 N4 Se2	C18 H8 F4 I2 N4 Se2
Mr	768.00	768.00
Dx,g cm-3	2.383	2.383
Z	16	16
Mu (mm-1)	6.386	6.386
F000	5664.0	5664.0
F000'	5648.82	
h,k,lmax	19,20,50	19,19,49
Nref	10883	9660
Tmin,Tmax	0.290,0.384	0.608,1.000
Tmin'	0.268	

Correction method= # Reported T Limits: Tmin=0.608 Tmax=1.000
AbsCorr = MULTI-SCAN

Data completeness= 0.888   Theta(max)= 28.532

R(reflections)= 0.0606(5900)   wR2(reflections)= 0.0880(9660)

S = 1.094   Npar= 541

The following ALERTS were generated. Each ALERT has the format
test-name_ALERT_alert-type_alert-level.
Click on the hyperlinks for more details of the test.

Alert level B

```
PLAT910_ALERT_3_B Missing # of FCF Reflection(s) Below Theta(Min).         17 Note
                 2  0  0,   2  1  0,   0  2  0,   1  1  1,   2  1  1,   0  2  1,
                 1  2  1,   0  0  2,   1  0  2,   2  0  2,   1  1  2,   0  2  2,
                 1  1  3,   0  2  3,   0  0  4,   1  0  4,   1  1  4,
```

Author Response: The missing FCF reflections below theta(min) were caused by the high beamstop theta(min) limit set.

Alert level C

```
PLAT334_ALERT_2_C Small <C-C> Benzene Dist.   C1       -C6       .       1.37 Ang.
PLAT342_ALERT_3_C Low Bond Precision on  C-C Bonds ...............    0.01014 Ang.
PLAT906_ALERT_3_C Large K Value in the Analysis of Variance ......     27.952 Check
PLAT906_ALERT_3_C Large K Value in the Analysis of Variance ......      4.836 Check
PLAT906_ALERT_3_C Large K Value in the Analysis of Variance ......      2.223 Check
PLAT911_ALERT_3_C Missing FCF Refl Between Thmin & STh/L=    0.600        119 Report
                 0 12  0,   0 14  0,   2 14  0,   2 15  0,   0 16  0,   2 16  0,
                 4 16  0,   2 17  0,   4 17  0,   0 18  0,   2 18  0,   4 18  0,
                 0 12  1,   1 13  1,   0 14  1,   1 14  1,   1 15  1,   2 15  1,
                 0 16  1,   1 16  1,   2 16  1,   3 16  1,   1 17  1,   2 17  1,
                 3 17  1,   4 17  1,   0 18  1,   1 18  1,   2 18  1,   3 18  1,
                 4 18  1,   0 12  2,   0 14  2,   1 14  2,   1 15  2,   2 15  2,
                 0 16  2,   1 16  2,   2 16  2,   3 16  2,   1 17  2,   2 17  2,
                 3 17  2,   4 17  2,   0 18  2,   1 18  2,   2 18  2,   3 18  2,
                 4 18  2,   0 14  3,   1 14  3,   1 15  3,   0 16  3,   1 16  3,
                 2 16  3,   1 17  3,   2 17  3,   3 17  3,   0 18  3,   1 18  3,
                 2 18  3,   3 18  3,   4 18  3,   0 14  4,   1 15  4,   0 16  4,
                 1 16  4,   2 16  4,   1 17  4,   2 17  4,   3 17  4,   0 18  4,
                 1 18  4,   2 18  4,   3 18  4,   4 18  4,   0 14  5,   0 16  5,
                 1 16  5,   1 17  5,   2 17  5,   0 18  5,   1 18  5,   2 18  5,
                 3 18  5,   0 16  6,   1 16  6,   1 17  6,   2 17  6,   0 18  6,
                 1 18  6,   2 18  6,   3 18  6,   0 16  7,   1 17  7,   0 18  7,
```

Alert level G

```
PLAT012_ALERT_1_G N.O.K.   _shelx_res_checksum Found in CIF ......     Please Check
PLAT013_ALERT_1_G N.O.K.   _shelx_hkl_checksum Found in CIF ......     Please Check
PLAT083_ALERT_2_G SHELXL Second Parameter in WGHT  Unusually Large       8.01 Why ?
PLAT431_ALERT_2_G Short Inter HL..A Contact  I1        ..N1       .       3.10 Ang.
                                                  x,y,z  =     1_555 Check
PLAT431_ALERT_2_G Short Inter HL..A Contact  I2        ..N3       .       3.05 Ang.
                                          x,3/2-y,1/2+z  =     7_576 Check
PLAT431_ALERT_2_G Short Inter HL..A Contact  I3        ..N5       .       3.04 Ang.
                                                  x,y,z  =     1_555 Check
PLAT431_ALERT_2_G Short Inter HL..A Contact  I4        ..N8       .       3.12 Ang.
                                         x,1/2-y,-1/2+z  =     7_565 Check
PLAT434_ALERT_2_G Short Inter HL..HL Contact F4        ..F8       .       2.80 Ang.
```

```
                                                      x,y,z  =      1_555 Check
PLAT434_ALERT_2_G Short Inter HL..HL Contact F6       ..F6       .       2.77 Ang.
                                                1-x,1-y,1-z  =      5_666 Check
PLAT912_ALERT_4_G Missing # of FCF Reflections Above STh/L=  0.600       1015 Note
PLAT969_ALERT_5_G The 'Henn et al.' R-Factor-gap value ..........        1.93 Note
              Predicted wR2: Based on SigI**2  4.56 or SHELX Weight  8.28
PLAT978_ALERT_2_G Number C-C Bonds with Positive Residual Density.          0 Info
```

```
   0 ALERT level A = Most likely a serious problem - resolve or explain
   1 ALERT level B = A potentially serious problem, consider carefully
   6 ALERT level C = Check. Ensure it is not caused by an omission or oversight
  12 ALERT level G = General information/check it is not something unexpected

   2 ALERT type 1 CIF construction/syntax error, inconsistent or missing data
   9 ALERT type 2 Indicator that the structure model may be wrong or deficient
   6 ALERT type 3 Indicator that the structure quality may be low
   1 ALERT type 4 Improvement, methodology, query or suggestion
   1 ALERT type 5 Informative message, check
```

It is advisable to attempt to resolve as many as possible of the alerts in all categories. Often the minor alerts point to easily fixed oversights, errors and omissions in your CIF or refinement strategy, so attention to these fine details can be worthwhile. In order to resolve some of the more serious problems it may be necessary to carry out additional measurements or structure refinements. However, the purpose of your study may justify the reported deviations and the more serious of these should normally be commented upon in the discussion or experimental section of a paper or in the "special_details" fields of the CIF. checkCIF was carefully designed to identify outliers and unusual parameters, but every test has its limitations and alerts that are not important in a particular case may appear. Conversely, the absence of alerts does not guarantee there are no aspects of the results needing attention. It is up to the individual to critically assess their own results and, if necessary, seek expert advice.

Publication of your CIF in IUCr journals

A basic structural check has been run on your CIF. These basic checks will be run on all CIFs submitted for publication in IUCr journals (*Acta Crystallographica*, *Journal of Applied Crystallography*, *Journal of Synchrotron Radiation*); however, if you intend to submit to *Acta Crystallographica Section C* or *E* or *IUCrData*, you should make sure that full publication checks are run on the final version of your CIF prior to submission.

Publication of your CIF in other journals

Please refer to the *Notes for Authors* of the relevant journal for any special instructions relating to CIF submission.

PLATON version of 06/01/2024; check.def file version of 05/01/2024

Datablock 2239823 - ellipsoid plot

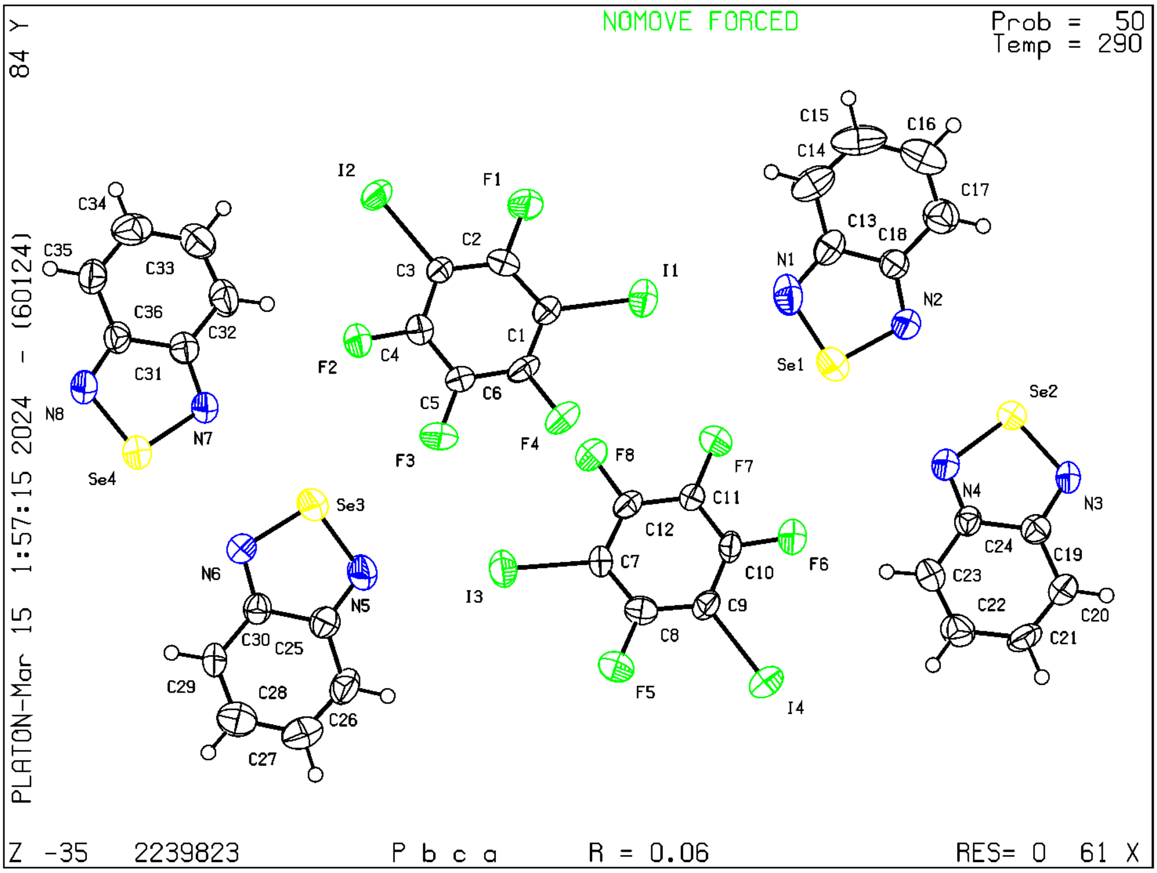

checkCIF/PLATON report

Structure factors have been supplied for datablock(s) 2239824

THIS REPORT IS FOR GUIDANCE ONLY. IF USED AS PART OF A REVIEW PROCEDURE FOR PUBLICATION, IT SHOULD NOT REPLACE THE EXPERTISE OF AN EXPERIENCED CRYSTALLOGRAPHIC REFEREE.

No syntax errors found. CIF dictionary Interpreting this report

Datablock: 2239824

Bond precision:	C-C = 0.0177 A		Wavelength=0.71073
Cell:	a=4.4302(2)	b=29.9768(14)	c=12.2962(7)
	alpha=90	beta=91.505(5)	gamma=90
Temperature:	293 K		

	Calculated	Reported
Volume	1632.41(14)	1632.41(14)
Space group	P 21/c	P 1 21/c 1
Hall group	-P 2ybc	-P 2ybc
Moiety formula	C6 F3 I3, C6 H4 N2 Se	C6 F3 I3, C6 H4 N2 Se
Sum formula	C12 H4 F3 I3 N2 Se	C12 H4 F3 I3 N2 Se
Mr	692.83	692.83
Dx,g cm-3	2.819	2.819
Z	4	4
Mu (mm-1)	7.997	7.997
F000	1240.0	1240.0
F000’	1234.61	
h,k,lmax	5,40,16	5,39,16
Nref	4134	3668
Tmin,Tmax	0.194,0.301	0.741,1.000
Tmin’	0.158	

Correction method= # Reported T Limits: Tmin=0.741 Tmax=1.000
AbsCorr = MULTI-SCAN

Data completeness= 0.887 Theta(max)= 28.537

R(reflections)= 0.0770(3132) wR2(reflections)= 0.1286(3668)

S = 1.308 Npar= 190

The following ALERTS were generated. Each ALERT has the format
test-name_ALERT_alert-type_alert-level.
Click on the hyperlinks for more details of the test.

Alert level C

```
PLAT334_ALERT_2_C Small <C-C> Benzene Dist.   C7       -C12      .       1.37 Ang.
PLAT342_ALERT_3_C Low Bond Precision on  C-C Bonds ..............    0.01775 Ang.
PLAT906_ALERT_3_C Large K Value in the Analysis of Variance ......     22.176 Check
PLAT906_ALERT_3_C Large K Value in the Analysis of Variance ......      3.961 Check
PLAT910_ALERT_3_C Missing # of FCF Reflection(s) Below Theta(Min).          6 Note
                0  2  0,    0  4  0,    0  1  1,    0  2  1,    0  3  1,    0  4  1,
PLAT971_ALERT_2_C Check Calcd Resid. Dens.  0.95Ang From I1             1.62 eA-3
```

Alert level G

```
PLAT012_ALERT_1_G No       _shelx_res_checksum Found in CIF ......     Please Check
PLAT083_ALERT_2_G SHELXL Second Parameter in WGHT  Unusually Large      24.45 Why ?
PLAT199_ALERT_1_G Reported _cell_measurement_temperature ..... (K)        293 Check
PLAT200_ALERT_1_G Reported   _diffrn_ambient_temperature ..... (K)        293 Check
PLAT431_ALERT_2_G Short Inter HL..A Contact  I1       ..N2       .       3.10 Ang.
                                                   x,y,z  =      1_555 Check
PLAT912_ALERT_4_G Missing # of FCF Reflections Above STh/L=  0.600        457 Note
PLAT969_ALERT_5_G The 'Henn et al.' R-Factor-gap value ..........       3.27 Note
              Predicted wR2: Based on SigI**2  3.94 or SHELX Weight 10.10
PLAT978_ALERT_2_G Number C-C Bonds with Positive Residual Density.          0 Info
```

0 **ALERT level A** = Most likely a serious problem - resolve or explain
0 **ALERT level B** = A potentially serious problem, consider carefully
6 **ALERT level C** = Check. Ensure it is not caused by an omission or oversight
8 **ALERT level G** = General information/check it is not something unexpected

3 ALERT type 1 CIF construction/syntax error, inconsistent or missing data
5 ALERT type 2 Indicator that the structure model may be wrong or deficient
4 ALERT type 3 Indicator that the structure quality may be low
1 ALERT type 4 Improvement, methodology, query or suggestion
1 ALERT type 5 Informative message, check

It is advisable to attempt to resolve as many as possible of the alerts in all categories. Often the minor alerts point to easily fixed oversights, errors and omissions in your CIF or refinement strategy, so attention to these fine details can be worthwhile. In order to resolve some of the more serious problems it may be necessary to carry out additional measurements or structure refinements. However, the purpose of your study may justify the reported deviations and the more serious of these should normally be commented upon in the discussion or experimental section of a paper or in the "special_details" fields of the CIF. checkCIF was carefully designed to identify outliers and unusual parameters, but every test has its limitations and alerts that are not important in a particular case may appear. Conversely, the absence of alerts does not guarantee there are no aspects of the results needing attention. It is up to the individual to critically assess their own results and, if necessary, seek expert advice.

Publication of your CIF in IUCr journals

A basic structural check has been run on your CIF. These basic checks will be run on all CIFs submitted for publication in IUCr journals (*Acta Crystallographica*, *Journal of Applied Crystallography*, *Journal of Synchrotron Radiation*); however, if you intend to submit to *Acta Crystallographica Section C* or *E* or *IUCrData*, you should make sure that full publication checks are run on the final version of your CIF prior to submission.

Publication of your CIF in other journals

Please refer to the *Notes for Authors* of the relevant journal for any special instructions relating to CIF submission.

PLATON version of 06/01/2024; check.def file version of 05/01/2024

Datablock 2239824 - ellipsoid plot

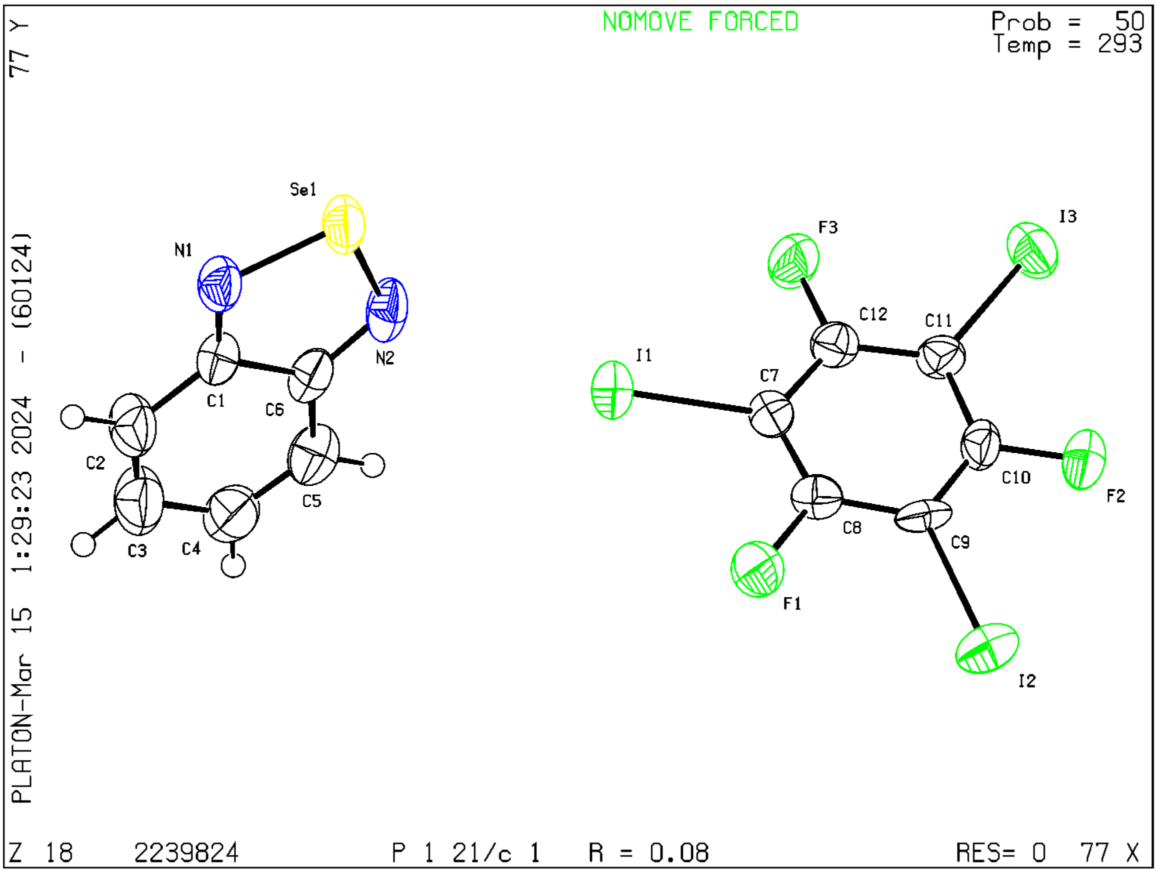

checkCIF/PLATON report

Structure factors have been supplied for datablock(s) 2417680

THIS REPORT IS FOR GUIDANCE ONLY. IF USED AS PART OF A REVIEW PROCEDURE FOR PUBLICATION, IT SHOULD NOT REPLACE THE EXPERTISE OF AN EXPERIENCED CRYSTALLOGRAPHIC REFEREE.

No syntax errors found.   CIF dictionary   Interpreting this report

Datablock: 2417680

```
Bond precision:   C-C = 0.0059 A          Wavelength=0.71073

Cell:             a=13.0258(6)     b=6.3844(3)          c=13.4476(6)
                  alpha=90         beta=104.852(5)      gamma=90
Temperature:      293 K
```

	Calculated	Reported
Volume	1080.97(9)	1080.97(9)
Space group	P 21/n	P 1 21/n 1
Hall group	-P 2yn	-P 2yn
Moiety formula	C6 F4 I2, 2(C6 H3 F N2 Se)	C6 F4 I2, 2(C6 H3 F N2 Se)
Sum formula	C18 H6 F6 I2 N4 Se2	C18 H6 F6 I2 N4 Se2
Mr	803.99	803.99
Dx,g cm-3	2.470	2.470
Z	2	2
Mu (mm-1)	6.342	6.342
F000	740.0	740.0
F000'	738.16	
h,k,lmax	17,8,18	17,8,18
Nref	2979	2678
Tmin,Tmax	0.163,0.319	0.551,1.000
Tmin'	0.113	

```
Correction method= # Reported T Limits: Tmin=0.551 Tmax=1.000
AbsCorr = MULTI-SCAN

Data completeness= 0.899          Theta(max)= 29.395

R(reflections)= 0.0349( 2002)           wR2(reflections)= 0.0626( 2678)

S = 1.036                  Npar= 145
```

The following ALERTS were generated. Each ALERT has the format
test-name_ALERT_alert-type_alert-level.
Click on the hyperlinks for more details of the test.

Alert level C

```
PLAT906_ALERT_3_C Large K Value in the Analysis of Variance ......      6.891 Check
```

Alert level G

```
PLAT012_ALERT_1_G N.O.K.   _shelx_res_checksum Found in CIF ......     Please Check
PLAT013_ALERT_1_G N.O.K.   _shelx_hkl_checksum Found in CIF ......     Please Check
PLAT199_ALERT_1_G Reported _cell_measurement_temperature ..... (K)        293 Check
PLAT200_ALERT_1_G Reported   _diffrn_ambient_temperature ..... (K)        293 Check
PLAT431_ALERT_2_G Short Inter HL..A Contact  I1       ..N2       .       2.99 Ang.
                                                      x,y,z  =      1_555 Check
PLAT910_ALERT_3_G Missing # of FCF Reflection(s) Below Theta(Min).          4 Note
                2  0  0,  -1  0  1,   1  0  1,   0  0  2,
PLAT912_ALERT_4_G Missing # of FCF Reflections Above STh/L=  0.600        297 Note
PLAT969_ALERT_5_G The 'Henn et al.' R-Factor-gap value ..........      1.326 Note
              Predicted wR2: Based on SigI**2  4.72 or SHELX Weight  6.04
PLAT978_ALERT_2_G Number C-C Bonds with Positive Residual Density.          3 Info
```

0 **ALERT level A** = Most likely a serious problem - resolve or explain
0 **ALERT level B** = A potentially serious problem, consider carefully
1 **ALERT level C** = Check. Ensure it is not caused by an omission or oversight
9 **ALERT level G** = General information/check it is not something unexpected

4 ALERT type 1 CIF construction/syntax error, inconsistent or missing data
2 ALERT type 2 Indicator that the structure model may be wrong or deficient
2 ALERT type 3 Indicator that the structure quality may be low
1 ALERT type 4 Improvement, methodology, query or suggestion
1 ALERT type 5 Informative message, check

It is advisable to attempt to resolve as many as possible of the alerts in all categories. Often the minor alerts point to easily fixed oversights, errors and omissions in your CIF or refinement strategy, so attention to these fine details can be worthwhile. In order to resolve some of the more serious problems it may be necessary to carry out additional measurements or structure refinements. However, the purpose of your study may justify the reported deviations and the more serious of these should normally be commented upon in the discussion or experimental section of a paper or in the "special_details" fields of the CIF. checkCIF was carefully designed to identify outliers and unusual parameters, but every test has its limitations and alerts that are not important in a particular case may appear. Conversely, the absence of alerts does not guarantee there are no aspects of the results needing attention. It is up to the individual to critically assess their own results and, if necessary, seek expert advice.

Publication of your CIF in IUCr journals

A basic structural check has been run on your CIF. These basic checks will be run on all CIFs submitted for publication in IUCr journals (*Acta Crystallographica*, *Journal of Applied Crystallography*, *Journal of Synchrotron Radiation*); however, if you intend to submit to *Acta Crystallographica Section C* or *E* or *IUCrData*, you should make sure that full publication checks are run on the final version of your CIF prior to submission.

Publication of your CIF in other journals

Please refer to the *Notes for Authors* of the relevant journal for any special instructions relating to CIF submission.

PLATON version of 19/12/2024; check.def file version of 19/12/2024

Datablock 2417680 - ellipsoid plot

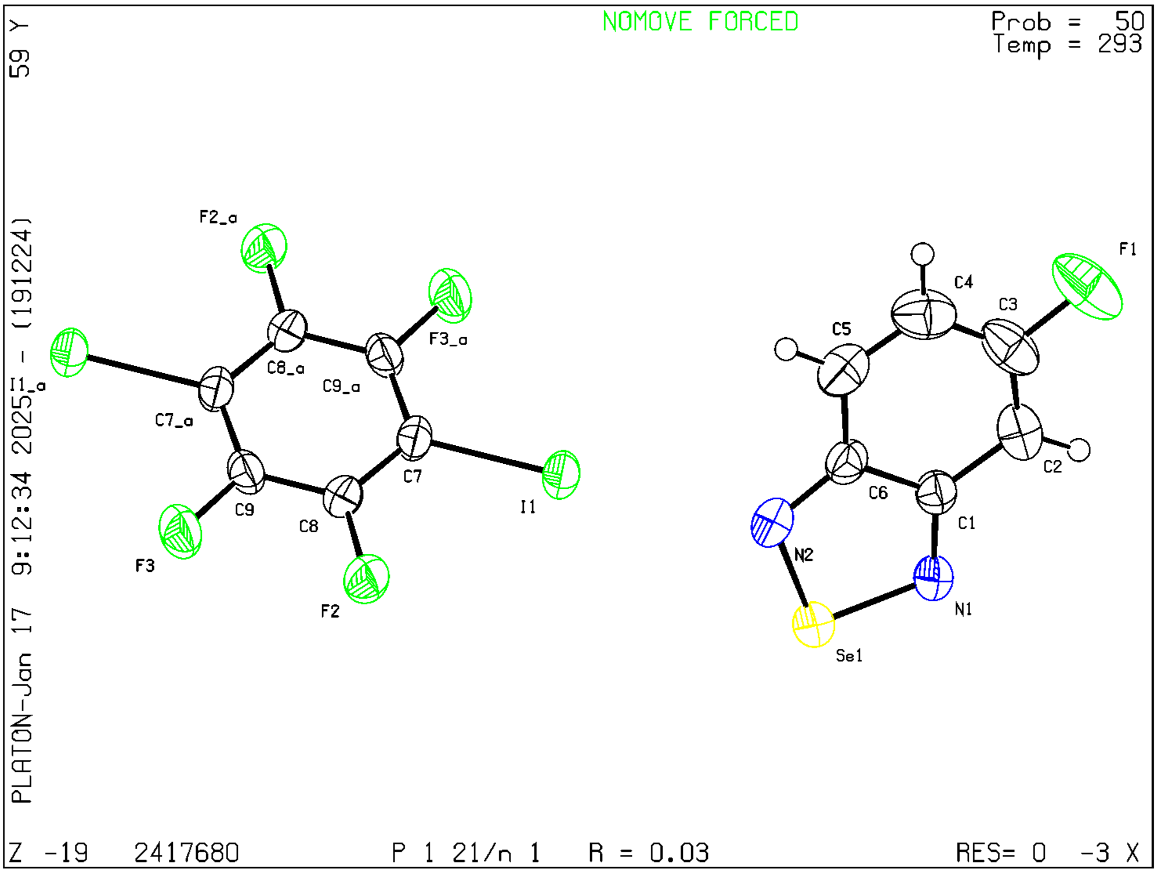

checkCIF/PLATON report

Structure factors have been supplied for datablock(s) 2417681

THIS REPORT IS FOR GUIDANCE ONLY. IF USED AS PART OF A REVIEW PROCEDURE FOR PUBLICATION, IT SHOULD NOT REPLACE THE EXPERTISE OF AN EXPERIENCED CRYSTALLOGRAPHIC REFEREE.

No syntax errors found. CIF dictionary Interpreting this report

Datablock: 2417681

Bond precision: C-C = 0.0125 A Wavelength=0.71073

Cell:	a=14.3599(6)	b=9.2659(4)	c=17.4527(9)
	alpha=90	beta=101.082(4)	gamma=90
Temperature:	100 K		

	Calculated	Reported
Volume	2278.91(18)	2278.91(18)
Space group	C 2/c	C 1 2/c 1
Hall group	-C 2yc	-C 2yc
Moiety formula	C6 F3 I3, 2(C6 H3 F N2 Se)	C6 F3 I3, 2(C6 H3 F N2 Se)
Sum formula	C18 H6 F5 I3 N4 Se2	C18 H6 F5 I3 N4 Se2
Mr	911.89	911.89
Dx,g cm-3	2.658	2.658
Z	4	4
Mu (mm-1)	7.364	7.364
F000	1656.0	1656.0
F000'	1650.48	
h,k,lmax	17,11,20	17,11,20
Nref	2017	2009
Tmin,Tmax	0.399,0.479	0.274,1.000
Tmin'	0.369	

Correction method= # Reported T Limits: Tmin=0.274 Tmax=1.000
AbsCorr = MULTI-SCAN

Data completeness= 0.996 Theta(max)= 25.027

R(reflections)= 0.0674(1865) wR2(reflections)= 0.1857(2009)

S = 1.113 Npar= 142

The following ALERTS were generated. Each ALERT has the format
test-name_ALERT_alert-type_alert-level.
Click on the hyperlinks for more details of the test.

Alert level C

```
DIFMX02_ALERT_1_C  The maximum difference density is > 0.1*ZMAX*0.75
            The relevant atom site should be identified.
PLAT097_ALERT_2_C Large Reported Max.  (Positive) Residual Density       4.20 eA-3
PLAT342_ALERT_3_C Low Bond Precision on  C-C Bonds ..............     0.0125 Ang.
PLAT906_ALERT_3_C Large K Value in the Analysis of Variance ......      3.334 Check
PLAT911_ALERT_3_C Missing FCF Refl Between Thmin & STh/L=    0.595          9 Report
                2  4  0,    8  6 11,    8  6 12,    7  1 15,    7  1 16,    8  0 16,
               -4  0 18,   -4  0 20,   -2  0 20,
```

Alert level G

```
PLAT003_ALERT_2_G Number of Uiso or U(i,j)  Restrained non-H-Atoms          4 Report
PLAT012_ALERT_1_G N.O.K.   _shelx_res_checksum Found in CIF ......     Please Check
PLAT072_ALERT_2_G SHELXL First  Parameter in WGHT  Unusually Large       0.13 Report
PLAT083_ALERT_2_G SHELXL Second Parameter in WGHT  Unusually Large       5.41 Why ?
PLAT171_ALERT_4_G The CIF-Embedded .res File Contains EADP Records          1 Report
PLAT186_ALERT_4_G The CIF-Embedded .res File Contains ISOR Records          2 Report
PLAT431_ALERT_2_G Short Inter HL..A Contact  I2        ..N2        .       3.01 Ang.
                                         -1/2+x,-1/2+y,z  =      3_445 Check
PLAT434_ALERT_2_G Short Inter HL..HL Contact I1        ..F2        .       3.07 Ang.
                                                 x,1+y,z  =      1_565 Check
PLAT720_ALERT_4_G Number of Unusual/Non-Standard Labels .........          1 Note
              F006
PLAT789_ALERT_4_G Atoms with Negative _atom_site_disorder_group  #          3 Check
PLAT822_ALERT_4_G CIF-embedded .res Contains Negative PART Numbers          2 Check
PLAT860_ALERT_3_G Number of Least-Squares Restraints ............          24 Note
PLAT870_ALERT_4_G ALERTS Related to Twinning Effects Suppressed ..          ! Info
PLAT909_ALERT_3_G Percentage of I>2sig(I) Data at Theta(Max) Still        85% Note
PLAT931_ALERT_5_G CIFcalcFCF  Twin Law ( 1 0 2)          Est.d BASF       0.59 Check
PLAT931_ALERT_5_G CIFcalcFCF  Twin Law          [ 2 5-1] Est.d BASF       0.56 Check
PLAT941_ALERT_3_G Average HKL Measurement Multiplicity ..........         1.0 Low
PLAT969_ALERT_5_G The 'Henn et al.' R-Factor-gap value ..........      3.048 Note
              Predicted wR2: Based on SigI**2  6.09 or SHELX Weight 16.67
```

0 **ALERT level A** = Most likely a serious problem - resolve or explain
0 **ALERT level B** = A potentially serious problem, consider carefully
5 **ALERT level C** = Check. Ensure it is not caused by an omission or oversight
18 **ALERT level G** = General information/check it is not something unexpected

2 ALERT type 1 CIF construction/syntax error, inconsistent or missing data
6 ALERT type 2 Indicator that the structure model may be wrong or deficient
6 ALERT type 3 Indicator that the structure quality may be low
6 ALERT type 4 Improvement, methodology, query or suggestion
3 ALERT type 5 Informative message, check

It is advisable to attempt to resolve as many as possible of the alerts in all categories. Often the minor alerts point to easily fixed oversights, errors and omissions in your CIF or refinement strategy, so attention to these fine details can be worthwhile. In order to resolve some of the more serious problems it may be necessary to carry out additional measurements or structure refinements. However, the purpose of your study may justify the reported deviations and the more serious of these should normally be commented upon in the discussion or experimental section of a paper or in the "special_details" fields of the CIF. checkCIF was carefully designed to identify outliers and unusual parameters, but every test has its limitations and alerts that are not important in a particular case may appear. Conversely, the absence of alerts does not guarantee there are no aspects of the results needing attention. It is up to the individual to critically assess their own results and, if necessary, seek expert advice.

Publication of your CIF in IUCr journals

A basic structural check has been run on your CIF. These basic checks will be run on all CIFs submitted for publication in IUCr journals (*Acta Crystallographica*, *Journal of Applied Crystallography*, *Journal of Synchrotron Radiation*); however, if you intend to submit to *Acta Crystallographica Section C* or *E* or *IUCrData*, you should make sure that full publication checks are run on the final version of your CIF prior to submission.

Publication of your CIF in other journals

Please refer to the *Notes for Authors* of the relevant journal for any special instructions relating to CIF submission.

PLATON version of 19/12/2024; check.def file version of 19/12/2024

Datablock 2417681 - ellipsoid plot

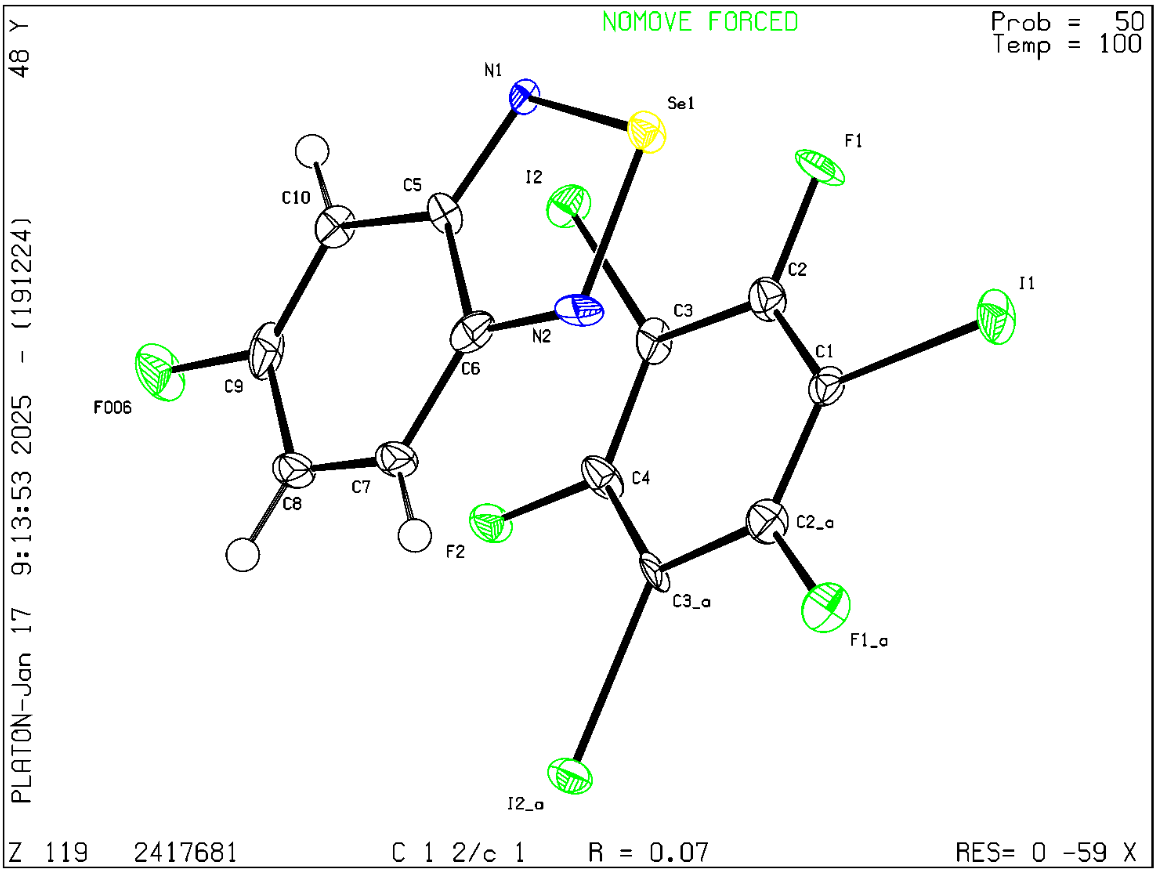

Supplement: Supplementary file 1 [file ijms-26-02324-s001.zip › Supplementary Materials/File S2.pdf]
